# Discovery of Intratumoral Oncolytic Bacteria Toward Targeted Anticancer Theranostics

**DOI:** 10.1002/advs.202301679

**Published:** 2023-05-07

**Authors:** Yamato Goto, Seigo Iwata, Mikako Miyahara, Eijiro Miyako

**Affiliations:** ^1^ Graduate School of Advanced Science and Technology Japan Advanced Institute of Science and Technology 1‐1 Asahidai Nomi Ishikawa 923‐1292 Japan

**Keywords:** bacteria, cancer, immunology, near‐infrared, theranostics

## Abstract

Unveiling biomedical functions of tumor‐resident microbiota is challenging for developing advanced anticancer medicines. This study demonstrates that isolated intratumoral bacteria, associated with natural purple photosynthetic bacteria, have inherent biocompatibility and strong immunogenic anticancer efficacies. They preferentially grow and proliferate within a targeted tumor milieu, which effectively causes immune cells to infiltrate the tumor and provoke strong anticancer responses in various syngeneic mouse models, including colorectal cancer, sarcoma, metastatic lung cancer, and extensive drug‐resistant breast cancer. Furthermore, these functional bacteria‐treated mice exhibit excellent anticancerous responses and have significantly prolonged survival rates with effective immunological memory. Light‐harvesting nanocomplexes of microbial consortia of intratumoral bacteria and purple photosynthetic bacteria can diagnose tumors using bio‐optical‐window near‐infrared light, making them useful theranostic agents for highly targeted immunological elimination of the tumor and for precisely marking tumor location.

## Graphical abstract

The authors isolate the microbial consortium AUN composed of *Proteus mirabilis* (A‐gyo) and *Rhodopseudomonas palustris* (UN‐gyo) from solid tumor biopsies. The functional bacteria AUN exhibits strong immunogenic anticancer efficacies against various tumor models. The AUN can also work as a tumor‐specific diagnosis probe. This research potentially provides an innovative cancer theranostics.

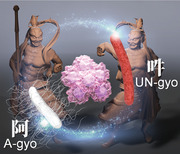

## 1 Introduction

Advanced anticancer medicines are highly effective against targeted tumors and are multifunctional, simplistic, safe, and inexpensive. Such treatments are essential for patients who experience medicinal side effects and financial issues related to typical cancer therapies.^[^ ^1^ ^]^ Bacterial cancer therapies employed in clinical trials are attractive for targeted cancer elimination because bacteria have promising properties that make them practical candidates for the treatment.^[^ ^2^, ^3^, ^4^, ^5^, ^6^ ^]^ Although bacteria are attractive therapeutic agents, conventional cancer therapy typically requires genetic engineering techniques,^[^ ^7^, ^8^ ^]^ synthetic bioengineering,^[^ ^9^, ^10^ ^]^ and nanotechnology^[^ ^11^, ^12^, ^13^ ^]^ for bacterial attenuation and improved drug efficacy because originally pathogenic bacteria with low medicinal properties are used. Moreover, various natural bacteria have been unaccountably applied for cancer treatment by many researchers for more than a century.^[^ ^2^, ^3^, ^4^, ^5^, ^6^ ^]^ Nevertheless, we believe it is important to explore natural biocompatible bacteria with stronger innate efficacies and higher tumor specificity without necessitating genetic manipulations and nanoengineering.^[^ ^14^ ^]^

Tumor‐resident microbiota may be another approach for exploring novel, functional, natural bacterial anticancer therapy. Recent evidence suggests that intratumoral bacteria can influence the efficacy of cancer therapies.^[^ ^15^, ^16^ ^]^ However, most studies have mainly focused on the causal role of intratumoral bacteria in cancer development or on their presence reflecting anticancer efficacies of chemotherapy against tumors.^[^ ^17^, ^18^, ^19^, ^20^ ^]^ Physically extracted and isolated intratumoral bacteria have not been used directly as an anticancer therapeutic agent, although there is an unrevealed possibility of their use for cancer treatment.

We aimed to explore highly targeted cancer immunotherapeutic bacteria from the tumor‐resident microbiota and its association with natural photosynthetic bacteria. The isolated intratumoral oncolytic bacteria were assessed for their functional anticancer efficacies, such as tumor‐targeting ability and biocompatibility in various syngeneic mouse models, regardless of the presence or absence of immunogenicity in the tumor. We also analyzed the survival rates of mice with unique immunological memory. In a targeted tumor theranostic approach, unique optical properties of intratumoral bacteria and photosynthetic bacteria were also explored using tissue‐penetrating near‐infrared (NIR) light. This research will shed light on the ever‐evolving biology of tumors and provide a novel approach to cancer treatment in addition to the development of advanced optical precision medical devices.

## 2 Results and Discussion

### 2.1 Isolation of Highly Effective Anticancer Bacteria from Tumors

**Figure 1:**
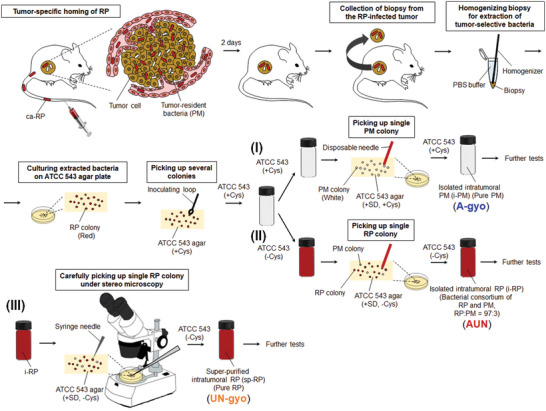
Schematic illustration of isolation of extremely effective anticancer bacteria from solid tumors. As the tumor‐specific homing of ca‐RP is an anaerobic process, ca‐RP can grow under anaerobic conditions in the tumor microenvironment (hypoxia). The scheme shows three approaches for the isolation of oncolytic bacteria, called A‐gyo, UN‐gyo, and AUN, from the colonies of intratumoral bacteria after intravenous administration of ca‐RP: (I) isolation process of A‐gyo using ATCC543 (+ Cys) liquid medium and ATCC543 (+ SD, + Cys) agar plate by conventional colony picking technique with a disposable needle, (II) isolation process of AUN using ATCC543 (‒ Cys) liquid medium and ATCC543 (+ SD, ‒ Cys) agar plate by conventional colony picking technique with a disposable needle, and (III) purification process of UN‐gyo from the bacterial consortium AUN (the mixture of A‐gyo and UN‐gyo) using ATCC543 (+ SD, ‒ Cys) medium and ATCC543 (+ SD, ‒ Cys) agar plate by microscopic colony picking technique with a sharp syringe needle.

Previously, we found that nonpathogenic natural purple photosynthetic bacteria, *Rhodopseudomonas palustris* (RP) and *Blastochloris viridis*, displayed multifunctionality (e.g., NIR‐I‐to‐NIR‐II reporter fluorescence, photothermal conversion, reactive oxygen species generation, and contrasting photoacoustic effects) and biocompatibility as cancer theranostic agents in the treatment of highly active cancers, using bio‐optical‐window I and II NIR light.^[^ ^14^ ^]^ A major challenge was the exploration of functional photosynthetic bacteria with superior anticancer therapeutic efficacy, which were accidentally discovered during the extractions of purple photosynthetic bacteria RP from its infected tumor biopsy for further characterization. A schematic illustration of the isolation of extremely effective anticancer bacteria from a solid tumor is depicted in **Figure** **1**. RP can colonize and grow in a tumor with high specificity because it prefers a hypoxic environment.^[^ ^13^ ^]^ We have previously reported characteristic red colonies of RP formed on an ATCC 543 agar plate after streaking the solution extracted from the RP‐infected tumor. Although conventional RP suspension is naturally red, the extracted solution from a solid tumor unexpectedly became gray over time because of the contamination with tumor‐resident microbiota.^[^ ^17^, ^18^ ^]^ *Proteus mirabilis* (PM) is anaerobic, gram‐negative, and a member of the intratumoral bacterial family *Enterobacteriaceae*.^[^ ^21^, ^22^ ^]^ Many white circular colonies of PM were formed on ATCC 543 agar plate with sodium deoxycholate (SD) that was used to culture single colonies as SD prohibits bacterial swarming (migration across surfaces of solid media) of PM.^[^ ^23^ ^]^ Isolated intratumoral PM (i‐PM) was confirmed to be 99.74% pure using gene identification (Table S1, Supporting Information). L‐cysteine (Cys) was used as a reductant agent to remove oxygen in ATCC 543 medium for effective culturing anaerobic bacteria. The addition of Cys unexpectedly promotes proliferation of i‐PM in the suspension with RP, resulting in color changing from red to gray, probably because an auxotrophic mutation in association with synthetic routes of Cys via transductive enzymatic reactions in i‐PM might be occurred by coculturing with RP.^[^ ^24^ ^]^

The solution of bacteria in the ATCC 543 medium without Cys turned red after subculturing and formed red and white colonies on an ATCC 543 agar plate with SD and without Cys, respectively. Although SD restraints bacterial swarming of PM, indistinct and translucent colonies of PM intermingle with RP colonies, making it difficult to sort a single colony of RP. Nevertheless, a single red colony of the intratumoral RP (i‐RP) was carefully picked up and cultured for further experiments. i‐RP was verified as an association of RP and PM by colony assays, fluorescent microscopy, and gene identification. The ratio of the microbial consortium was perpetually RP:PM = 97:3 even after repetitive subculturing, as confirmed using fluorescence microscopy and colony assays. These results potentially indicate that i‐PM has a symbiotic relationship (commensalism) with i‐RP due to the aforementioned mutation involved with Cys metabolism in i‐PM cell.

Moreover, super‐purified RP (sp‐RP) was successfully obtained from a PM‐swarmed single red colony of i‐RP using a fine‐tipped syringe needle (≈337 µm in diameter) under stereo microscopy. sp‐RP was identified as 100% pure RP using genetic tests (Table S2, Supporting Information).

Hereafter we present A‐gyo, UN‐gyo, and AUN, isolated intratumoral bacteria i‐PM, sp‐RP, and i‐RP, respectively.

### 2.2 Antitumor Efficacy of Functional Bacteria

**Figure 2:**
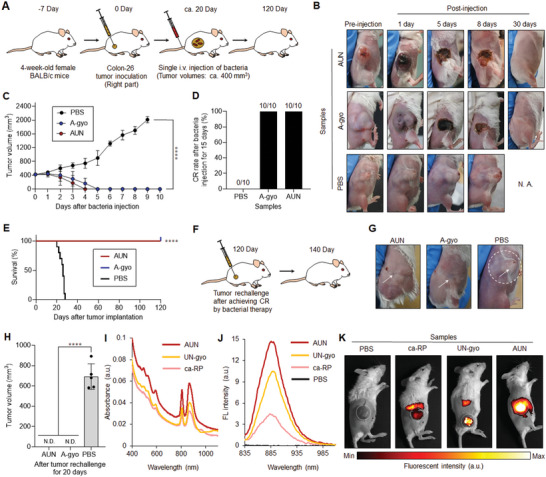
Bacteria‐based theranostics for Colon‐26‐tumor‐bearing mice. A) Schematic illustration of in vivo Colon‐26 carcinoma antitumor tests using various functional bacteria. B) Images of mice after each treatment. N. A., not available. C) In vivo anticancer effect of functional bacteria. The PBS or suspension of bacteria was intravenously injected into Colon‐26‐bearing mice. Data are represented as mean ± standard errors of the mean (SEM); *n* = 5 biologically independent mice. ****, *p* < 0.0001, by two‐way ANOVA test. D) Complete response (CR) rate of Colon‐26‐tumor‐bearing mice (*n* = 10 biologically independent mice) at day 15 after bacteria or PBS injection. E) Kaplan–Meier survival curves of Colon‐26‐tumor‐bearing mice (*n* = 10 biologically independent mice) after tumor implantation. Statistical significance was calculated by comparison with the PBS group. ****, *p* < 0.0001, Log‐rank (Mantel–Cox) test. F) Schematic illustration of tumor rechallenge study after bacterial cancer therapy. Second Colon‐26 tumor inoculation into mice (right side) after bacterial treatments or the control experiment with PBS injection is performed at day 120. G) Images of mice in each group after second tumor inoculations assessed after 20 days. The white arrows represent the position of tumor inoculation. The white dashed circle displays the location of the solid tumor. H) The tumor volumes for each treated mouse (*n* = 5 biologically independent mice) at day 20 after tumor inoculation. N. D., not detectable; ****, *p* < 0.0001, by Student's *t* two‐sided test. I) UV–vis–NIR absorbance of various RP suspensions. J) Fluorescent emission spectra of various RP suspensions excited at 805 nm. K) In vivo NIR fluorescent bio‐imaging of Colon‐26‐tumor‐bearing mice after intravenous injection of PBS (200 µL) or each RP suspension (ca‐RP, UN‐gyo, and AUN) (200 µL, 1 × 10^9^ CFU) for 5 days. White dashed circles represent the location of tumors.

In vivo antitumor therapeutic efficacy of isolated functional bacteria was investigated using a murine Colon‐26 carcinoma tumor syngeneic model (**Figure** **2**A). Tumor volumes were measured for 120 days after starting the therapeutic regime. Colon‐26‐bearing immunocompetent mice were injected intravenously (i.v.) with each bacterial suspension or PBS.

PBS injections did not influence tumor growth. Meanwhile, surprisingly, AUN, A‐gyo, and A‐gyo + UN‐gyo achieved dramatic tumor suppression and complete response (CR) after a single‐dose administration (Figure 2B–2D; and Figure S1, Supporting Information). Tumors completely disappeared after i.v. injection of AUN, A‐gyo, or A‐gyo + UN‐gyo, and provided an excellent prognosis with no recurrence after treatment. CR rates of AUN and A‐gyo were higher than that for A‐gyo + UN‐gyo because tumor recurrence was observed in the A‐gyo + UN‐gyo group after bacterial injections for 15 days. For accomplishing CR of tumors, optimal bacterial counts of AUN and A‐gyo were found to be 1 × 10^9^ and 1 × 10^8^ CFU, respectively (Figure S2A and S2B, Supporting Information). The i‐PM isolated from solid tumor biopsies without i.v. injection of ca‐RP has also the same anticancer efficacy as A‐gyo (Figure S2A, Supporting Information).

Although other bacteria, such as UN‐gyo and commercially available RP (ca‐RP), showed a partial tumor response, these groups were not as effective as AUN, A‐gyo, and A‐gyo + UN‐gyo (Figure S1, Supporting Information). This suggests that RP and PM's microbial consortium possesses anticancer efficacy compared with pure bacteria (UN‐gyo and ca‐RP), presumably because of such association that can lead to synergetic benefits due to bacterial cross‐talks such as biochemical reactions and interbacterial signaling.^[^ ^25^, ^26^ ^]^ Commensalism of A‐gyo derived from Cys against UN‐gyo might also cause immunogenic mutation of these bacteria in addition to formation of therapeutic metabolites to be a stronger immunological stimulant for effective tumor eradication. Anyway, further explorations such as whole genome analysis and metatranscriptomics are challenging to identify the detailed mechanism and relationship between A‐gyo and UN‐gyo.

Different strains of PM, such as PM Hauser (ATCC 35 659), are reported to possess oncolytic effects in murine tumors.^[^ ^27^ ^]^ However, the condition of mice deteriorated a day after administration with commercially available PM (ca‐PM) (NBRC 3849 and ATCC 35 659). Movement disorders (crouching position and shivering) and hypothermia were observed in the ca‐PM injected mice; hence further in vivo tests using ca‐PM were immediately terminated. This result indicated that A‐gyo could be attenuated while keeping strong anticancer efficacy because biocompatible A‐gyo was obtained from living mice. This is the first report showing that isolated intratumoral bacteria demonstrate dramatic CR to the tumor after a single administration of bacteria.

The survival rate of mice after i.v. injection of AUN or A‐gyo was significantly prolonged compared to the control groups (UN‐gyo, ca‐RP, and PBS) (*p* < 0.0001), showing a 100% survival after 120 days (Figure 2E; and Figure S1, Supporting Information). No significant weight loss was observed in mice, indicating that bacteria exhibit low systemic toxicity except for ca‐PM (Figure S3, Supporting Information).

To investigate the effect of bacterial treatment on immunological memory response in vivo, we used a tumor rechallenge model (tumor‐free mice surviving from a previous survival rate test and naive mice). Tumor developments after the first inoculation were conducted for CR‐achieved mice by treatment with AUN (the microbial consortium of A‐gyo and UN‐gyo) or A‐gyo on day 120 (Figure 2F). Interestingly, bacterial treatments could acquire substantial antitumor immunity in the Colon‐26 tumor model because of the recruitment of immune cells in the tumor microenvironment.^[^ ^28^, ^29^ ^]^ In effect, 100% of mice treated with AUN or A‐gyo successfully rejected tumor rechallenge (via subcutaneous injection of 1 × 10^6^ Colon‐26 cells), demonstrating that functional bacteria can induce durable antitumor immunity (Figure 2G,H). More surprisingly, these treated mice were healthy without tumor recurrence for more than 300 days in the experimental scheme, even after tumor rechallenge. Besides, the tumor rechallenge study of the PBS‐injected control mice revealed massive tumor formation. CD4^+^ and CD8^+^ memory T cells were increased in the A‐gyo‐ and AUN‐treated tumors; hence, we hypothesized that these memory T cells could have an inhibitory effect on tumor growth (Figure S4, Supporting Information).^[^ ^29^ ^]^ Flow cytometry analyses also said that these CD4^+^, CD8^+^, and CD45RO^+^ memory T cells were expressed in tumors after i.v. injection of A‐gyo and AUN for 3 and 24 h in comparison with PBS‐treated tumors (Figure S5, Supporting Information).

Therefore, an anticancer strategy using intratumoral bacteria and its microbial consortium could effectively kill primary tumors in response to injected external bacteria and elicit immunological memory by stimulating systemic antitumor immunity. The intratumoral bacterial platform proposed in this study might offer a new strategy for improving therapeutic outcomes and inhibiting tumor recurrence. These data suggest that functional bacteria could be useful for long‐term tumor‐specific protection.

We previously reported that ca‐RP suspensions have specific absorbance in deep tissue‐penetrable NIR region at around 805 and 850 nm derived from the light‐harvesting‐1‐reaction center (LH1‐RC) and peripheral light‐harvesting‐2 (LH2) antenna protein nanocomplexes.^[^ ^14^, ^30^ ^]^ The various RP solutions (AUN, UN‐gyo, and ca‐RP) prepared in this study displayed the characteristic NIR absorbance (Figure 2I) and fluorescence (FL) by NIR excitation at 805 nm (Figure 2J). Among them, AUN effectively absorbed NIR light and expressed the strongest NIR FL. In addition, in vivo bio‐imaging revealed significantly increased FL in all RP strains in targeted tumors (Figure 2K). Among them, NIR FL derived from tumor specificity of AUN was superior to that of ca‐RP and UN‐gyo. It is known that the tumor‐isolated strains present in a tumor growing in mice allow increased targeting of the tumor cells by the immune system.^[^ ^31^, ^32^ ^]^ Therefore, these strong optical properties of AUN might be attributed to the unique biological screening of tumor‐specific mutants obtained from a solid tumor surviving in a harsh environment compared to the external RP, resulting in excitometabolism of LH1‐RC and LH2 nanocomplexes. Indeed, the microbial consortium of AUN might also improve the optical properties because bacterial communications are known to facilitate metabolism.^[^ ^25^, ^26^ ^]^ Meanwhile, NIR FL derived from AUN was still observed in liver even after achieving CR of tumors at Day 5, Day 7, and Day 10 due to the BChl residues in “deactivated” bacteria by immune cells^[^ ^14^ ^]^ (Figure S6A, Supporting Information). We confirmed that the extractions from vital organs did not form “live” bacterial colonies of AUN on agar plates after i.v. injection of AUN for 7 days (Figure S6B, Supporting Information). In any case, these results indicated that AUN could serve as a useful NIR FL probe in mice and be effective as a therapeutic agent for multidimensional tumor targeting and elimination.

### 2.3 Biological Distribution of Functional Bacteria in the Tumor

**Figure 3:**
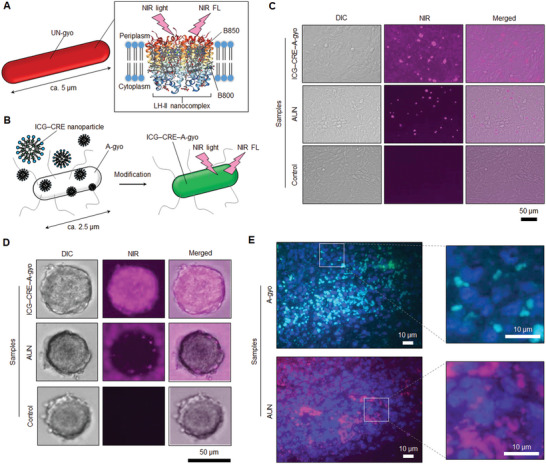
In vitro and in vivo biological distribution of bacteria in cancer cells, spheroids, and tissues. A) Schematic illustration of NIR light‐triggered NIR fluorescence (FL) detection of RP (UN‐gyo). B) Scheme of NIR fluorescent ICG‒CRE‒A‐gyo synthesis. C) FL images of live Colon‐26 cells after treatment with ICG‒CRE‒A‐gyo and AUN for 4 h at 37 °C. The pink FL is from bacteria. D) Fluorescent imaging of cancer spheroids incubated with ICG‒CRE‒A‐gyo and AUN for 24 h to evaluate tumor penetration ability. E) Observation of bacterial distribution in solid tumor tissue by FISH analysis. Bacterial cells and colonies of A‐gyo and AUN are light blue and pink, respectively. Cancer cells (blue) were counterstained with 4',6‐diamidino‐2‐phenylindole (DAPI).

The biological distribution of functional bacteria in cancer cells, spheroids, and tumor tissues was investigated to clarify the tumor penetration and eradication capacity of the AUN and A‐gyo (**Figure** **3**). Fluorescent microscopy was used for the convenient visualization of these properties of bacteria. Although AUN has naturally strong NIR FL derived from LH1‐RC and LH2 nanocomplexes (Figure 3A), A‐gyo does not possess FL in any region to explore their properties using fluorescence microscopy (Figure S7, Supporting Information). Thus, the nanoengineering method using indocyanine green‐encapsulating Cremophor EL (ICG‒CRE) nanoparticle was first applied to donate NIR FL to A‐gyo (Figure 3B).^[^ ^33^ ^]^ Synthesized ICG‒CRE modified A‐gyo (ICG‒CRE‒A‐gyo) exhibited unique optical absorbance and fluorescence properties (Figure S8, Supporting Information). The viability of A‐gyo could be kept at more than 90% during functionalization with ICG‒CRE nanoparticles. The content of the loading and the internalization of ICG was ≈140 µg mL^−1^ for 2.0 × 10^9^ CFU mL^−1^ of A‐gyo. ICG‒CRE nanoparticles were gradually exfoliated from the engineered bacteria over time under ambient temperature at 25 °C presumably because external molecules of ICG and CRE were excluded by bacterial efflux pumps utilizing cellular energy (Figure S9, Supporting Information).^[^ ^34^ ^]^ In fact, ICG‒CRE nanoparticles could be tightly adsorbed in A‐gyo at least 24 h under cold atmosphere at 4 °C where bacteria were not able to use cellular energy for the exclusion of intracellular molecules (Figure S9, Supporting Information).

The intracellular distribution of AUN and ICG‒CRE‒A‐gyo in Colon‐26 cells were evaluated using fluorescence microscopy, which revealed intracellular ICG‒CRE‒A‐gyo uptake in cells after incubation at 37 °C for 4 h (Figure 3C; and Figure S10, Supporting Information). NIR FL of ICG‒CRE‒A‐gyo (pink‐colored dots) proved its uniform distribution in the cell cytoplasm. Though AUN showed NIR FL in cells after incubation at the same condition, its intensity was not stronger than that of the cells harboring ICG‒CRE‒A‐gyo. A‐gyo is a highly motile peritrichously flagellated microbe, and an average length of A‐gyo (≈2.5 µm) is smaller than that of UN‐gyo (majority of bacteria in AUN; ≈5 µm), resulting in effective internalization of A‐gyo into cells. The exfoliated ICG‒CRE nanoparticles from ICG‒CRE‒A‐gyo might be also somewhat contributed on the intracellular fluorescent distribution. The control cells without bacterial treatment did not show any FL. Meanwhile, after incubating cells with both bacteria (AUN and ICG‒CRE‒A‐gyo) at 4 °C for 4 h, NIR FL assessed from bacteria was not strong, suggesting that the bacterial internalization into cells was energy dominant.^[^ ^35^ ^]^ Herein, endocytosis, size and shape of bacteria, and flagella‐driven motility are important factors for potential intracellular migrations of functional bacteria.

We further studied the tumor penetration capacity of the ICG‒CRE‒A‐gyo and AUN in a spheroid model that originated from Colon‐26 cancer cells (Figure 3D). Compact‐sized and highly motile ICG‒CRE‒A‐gyo could effectively enter tumor spheroids compared to AUN. This reveals that the mobility of bacteria aids in penetrating the core region of the solid tumor effectively.

However, it is difficult to explain the reason for the high tumor specificity of bacteria based on these in vitro “static” tests because both hypoxic environments of tumors and dynamic blood flow are principally essential for the cancer selectivity of bacteria. Various anaerobic bacteria can recognize the hypoxia microenvironment in a tumor after their systemic circulation in huge blood vessel networks in the body of a mouse. Thus, using microbial fluorescence in situ hybridization (FISH) assay,^[^ ^36^ ^]^ intratumoral distributions of A‐gyo and AUN cells and their colonies were observed in tumors after i.v. injections of A‐gyo and AUN through the tail vein of Colon‐26‐bearing mice (Figure 3E). The control (PBS injection) revealed that the native tumor‐resident PMs (green‐colored dots) were also present in a tumor. In contrast, red fluorescence derived from RP was not observed (Figure S11, Supporting Information). Herein, we conclude that A‐gyo and AUN can deeply penetrate a solid tumor with the help of blood flow and anaerobically proliferate in hypoxic tumor conditions.

### 2.4 Mechanism of Tumor Suppression by Functional Bacteria

**Figure 4:**
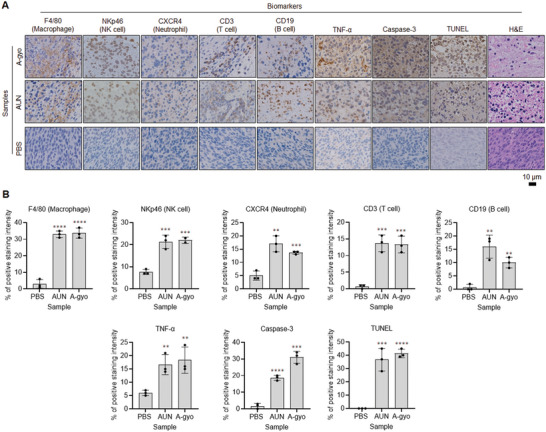
Mechanism of tumor suppression by functional bacteria. A) IHC (F4/80, NKp46, CXCR4, CD3, CD19, TNF‐*α*, and caspase‐3), TUNEL, and hematoxylin and eosin (H&E) stained tumor tissues collected from different groups of mice at day 1 after respective treatments (PBS, AUN, and A‐gyo). B) Statistical analyses of IHC (F4/80, NKp46, CXCR4, CD3, CD19, TNF‐*α* and caspase‐3) and TUNEL‐positive stained tumor tissues in (A). Data are represented as mean ± SEM; *n* = 3 independent areas (region of interest) in each tumor tissue collected from the groups of mice on day 1 after treatments with A‐gyo, AUN, and PBS. Statistical significance was calculated in comparison with the PBS group. *, *p* < 0.05, **, *p* < 0.01, ***, *p* < 0.001, and ****, *p* < 0.0001, by Student's *t* one‐sided test.

To investigate the immunological mechanism related to solid tumor suppression by bacterial injection, hematoxylin and eosin (H&E) and immunohistochemical (IHC) staining analyses were performed (**Figure** **4**; and Figure S12, Supporting Information). Cross‐sections of tumor tissue slices after IHC staining of F4/80 (macrophage marker), CXCR4 (neutrophil marker), NKp46 [natural killer cell marker], CD3 (T cell marker), and CD19 (B cell marker) could recognize the immunological reactions for the regulation of tumor suppression by functional bacteria. A‐gyo and AUN exhibited the expression of all immunological biomarkers. This indicates that the total mobilization of immune cells is activated in the tumor milieu by i.v. injection of A‐gyo and AUN. Another highly antitumor bacterial mixture (A‐gyo + UN‐gyo) significantly stimulates macrophage, and T and B cells are also aggressive immunological cells for tumor elimination. Other control bacterial groups of ca‐RP and UN‐gyo were ineffective for immunological activation compared to A‐gyo and AUN. PBS injection did not influence the expression of immunological biomarkers. We also confirmed that A‐gyo and AUN could evoke various immune cells in the tumor tissues after i.v. injection for 3 and/or 24 h, using flow cytometry analyses (Figure S13, Supporting Information).

In addition, tumor necrosis factor‐*α* (TNF‐*α*), a multifunctional cytokine secreted primarily by macrophages, was particularly expressed in A‐gyo and AUN groups. However, other bacterial groups indicated sufficient TNF‐*α* expression.

Caspase‐3 staining assay represented many apoptotic cells, especially in A‐gyo, AUN, and A‐gyo + UN‐gyo groups. Obvious caspase‐3 staining in tumor slices after treatments with these intratumoral functional bacteria and microbial consortium indicates massive apoptotic cell death evoked by immunological stimulation features from these bacteria. Simultaneously, terminal deoxynucleotidyl transferase (TdT)‐mediated 2´‐deoxyuridine, 5´‐triphosphate (dUTP) nick end labeling (TUNEL) assay was also commanded to support further strong in vivo anticancer mechanism for the intratumoral bacteria. Treatment of intratumoral bacteria (A‐gyo, AUN, and A‐gyo + UN‐gyo) enhanced cancer cell apoptosis, as indicated by increased TUNEL‐positive cells. Other control bacterial strains (ca‐RP and UN‐gyo) contributed to apoptotic TUNEL color development to a certain degree. In contrast, the control group of PBS alone did not exhibit apoptotic TUNEL color development within the tumor mass.

Functional bacteria A‐gyo, AUN, and A‐gyo + UN‐gyo groups demonstrated obvious tumor damage with intercellular fragmentation by H&E staining assay, further revealing antitumor effectiveness. Although other control bacterial injection groups (ca‐RP and UN‐gyo) also showed tumor degeneration, the antitumor therapeutic effectiveness was lower than that observed in the intratumoral bacteria A‐gyo and its microbial consortium of AUN and A‐gyo + UN‐gyo groups. On the other hand, healthy pathological features, such as tight arrangement and nuclear atypia, were observed in control PBS‐alone group.

**Figure 5:**
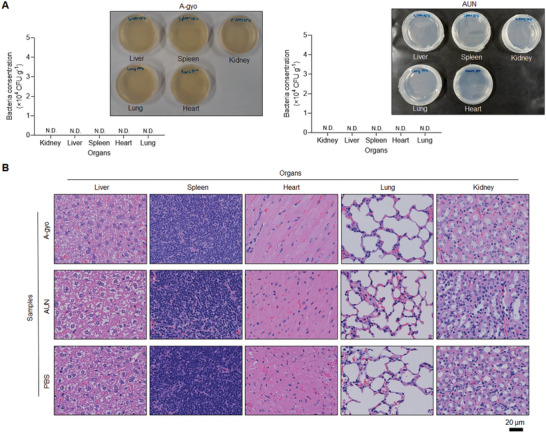
Biocompatibility of functional bacteria. A) Numbers and images of the bacterial colony of organs in Colon‐26‐tumor‐bearing mice after i.v. injection of A‐gyo (left) (200 µL, 1 × 10^8^ CFU) or AUN (right) (200 µL, 1 × 10^9^ CFU) after 25 days. Data are represented as mean ± SEM; *n* = 5 independent experiments. N. D., not detectable. B) H&E staining in conventional organs sectioned after i.v. injection of A‐gyo (left) (200 µL, 1 × 10^8^ CFU) or AUN (right) (200 µL, 1 × 10^9^ CFU) after 30 days.

No matter what aggressive immune responses the functional bacteria A‐gyo and AUN (a microbial consortium of A‐gyo and UN‐gyo) can evoke in the tumor microenvironment, we believe that it would not undergo immunological crisis in other healthy organs. These bacterial colonies were not detectable postinjection at day 25 in vital organs, such as the kidney, liver, spleen, heart, and lung, after treatments in Colon‐26‐bearing mice using colony counting assays (**Figure** **5**A). It indicates that functional bacteria simultaneously and naturally disappear after tumor elimination, making it unnecessary to prescribe an antibiotic.

Furthermore, functional bacteria A‐gyo and AUN did not show any toxicity in tissues after i.v. injection for 30 days (Figure 5B). H&E staining analyses demonstrated that the tissues of post i.v. injection of A‐gyo and AUN resemble that of control (PBS buffer).

The complete blood counts (CBCs) and blood biochemical parameters of mice were also evaluated after i.v. injection of PBS or bacterial suspension containing A‐gyo or AUN for 30 days (Tables S3 and S4, Supporting Information). No significant difference was observed between bacterial administration and control PBS‐injected mice groups. Thus, we conclude that the bacterial suspensions were not toxic for in vivo administration.

### 2.5 Therapeutic Extensibility of Functional Bacteria Against Various Cancer Types

**Figure 6:**
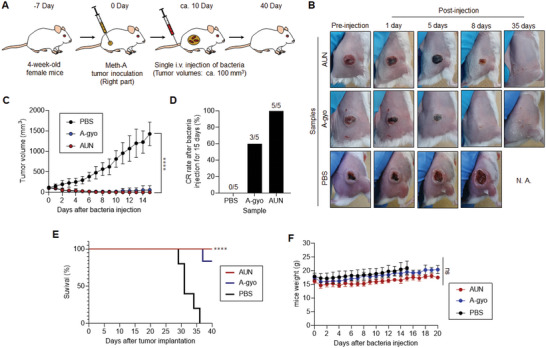
Antitumor efficacy of functional bacteria against sarcoma model. A) Schematic illustration of in vivo sarcoma antitumor tests using functional bacteria. B) Images of mice after each treatment. C) In vivo anticancer effect of functional bacteria. The PBS or suspension of bacteria was injected intravenously into Meth‐A‐bearing mice. Data are represented as mean ± SEM; *n* = 5 biologically independent mice. ****, *p* < 0.0001, by two‐way ANOVA test. D) CR rate of sarcoma‐bearing mice (*n* = 5 biologically independent mice) injected with bacteria, or PBS assessed after 15 days. E) Kaplan–Meier survival curves of sarcoma‐bearing mice (*n* = 5 biologically independent mice) after tumor implantation. Statistical significance was calculated in comparison with the PBS group. ****, *p* < 0.0001, Log‐rank (Mantel–Cox) test. F) Weight of mice after each treatment. Data are represented as means ± SEM; *n* = 5 independent experiments. ns, not significant, by two‐way ANOVA test.

Our bacterial cancer immunotherapies were designed to work with the immune system to increase native antitumor responses. The functional intratumoral bacteria and its microbial consortium were effective against immunogenic Colon‐26 malignant tumor. Finally, the presence of such functional bacteria was investigated in treatments of other cancer types with different immunogenicities (**Figure** **6**).

AUN particularly represented dramatic effectiveness against an immunogenic murine skin sarcoma (Meth‐A)‐bearing tumor model and accomplished 100% of the CR rate of tumors (Figure 6A–D). A‐gyo also exhibited high anticancer efficacy (CR rate = 60%), but tumor reoccurrences were observed around 10 days after achieving CR in some cases. Both AUN and A‐gyo improved the survival rate of Meth‐A‐bearing mice (Figure 6E). Furthermore, mortality rates were 0% after i.v. injections with AUN or A‐gyo after 40 days. There were no significant differences in the weight of mice between PBS and these bacterial injected groups, demonstrating that the bacteria did not have any side effects on Meth‐A‐bearing mice (Figure 6F).

**Figure 7:**
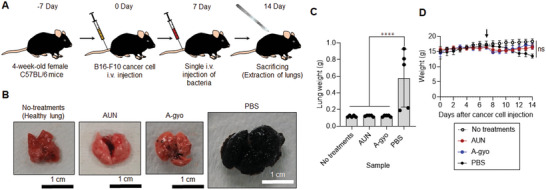
Antitumor efficacy of functional bacteria against metastatic lung cancer model. A) Schematic illustration of in vivo antitumor tests using functional bacteria against metastatic lung cancer model. B) Images of lungs after each treatment. C) Weight of lungs after each treatment. Data are represented as mean ± SEM; *n* = 5 independent experiments. ****, *p* < 0.0001, by two‐way ANOVA test. D) Average body weight of the mice after bacterial and control treatments during the experimental duration. The black arrow shows the time point of sample injection (Day 7). Data are represented as the mean ± SEM; *n* = 5 biologically independent mice. ns, not significant, by Student's *t* two‐sided test.

Interestingly, these bacteria also showed potent antitumor efficacy against metastatic lung cancer derived from nonimmunogenic murine melanoma (B16F10) (**Figure** **7**A–D). Lungs excised from the no‐treatment group exhibited normal physiological features, and the lungs of mice treated with AUN resembled those of controls after i.v. injection (Figure 7B). Although A‐gyo‐treated lungs had slightly metastatic pulmonary nodules (small black dots), they looked almost similar to native lungs. Meanwhile, the lungs of B16F10 melanoma‐bearing mice revealed enlarged black tumors. The lungs of mice treated with both (B16F10 implantation and AUN or A‐gyo i.v. injection) exhibited a significantly reduced tumor weight versus B16F10 melanoma‐bearing mice (Figure 7C). The average body weights of mice treated with B16F10 cells and AUN or A‐gyo were determined, and there were no significant differences compared to that of the control groups (no‐treatment and treatment with B16F10 cells and PBS) (Figure 7D).

**Figure 8:**
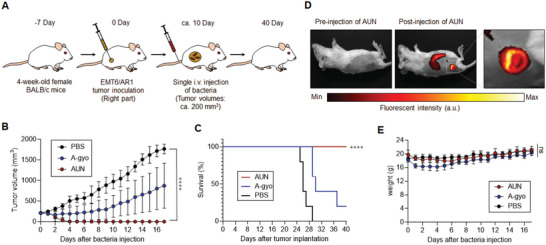
Antitumor efficacy of functional bacteria against drug‐resistant cancer model. A) Schematic illustration of in vivo antitumor tests using functional bacteria against drug‐resistant cancer model. B) Relative volumes of the tumors on the right flank of mice after i.v. injection of AUN, A‐gyo, or PBS. Bacterial concentrations of AUN and A‐gyo are 10 × 10^9^ and 5 × 10^8^ CFU mL^−1^, respectively. Data are presented as mean ± SEM (*n* = 5 biologically independent tests), ****, *p* < 0.0001 (Student's two‐sided *t*‐test used for analysis in comparison to PBS). C) Kaplan–Meier survival curves of EMT6/AR1‐bearing mice (*n* = 5 biologically independent mice) after tumor implantation. Statistical significance was calculated in comparison with the PBS group. ****, *p* < 0.0001, Log‐rank (Mantel‐Cox) test. D) NIR FL images of the mouse before and after i.v. injection of AUN (5 × 10^9^ CFU mL^−1^) for 3 days. E) Weight of mice after each treatment. Data are represented as means ± SEM; *n* = 5 independent experiments. ns, not significant, by Student's *t* two‐sided test.

Moreover, poorly immunogenic and drug‐resistant mouse mammary tumor (EMT6/AR1)‐bearing mice were also effectively cured by AUN treatment (**Figure** **8**A–E). AUN injection successfully achieved CR of tumors (Figure 8B; and Figure S14, Supporting Information). The single i.v. administration of AUN prolonged the survival rates of mice by at least 40 days (Figure 8C). Optical nanofunction of AUN was also useful for effective NIR FL marking of tumor location (Figure 8D). Meanwhile, A‐gyo represented a mild antitumor efficacy against the extensive drug‐resistant model (Figure 8D). Both bacteria were safe for EMT6/AR1‐bearing mice and other cancer models, resulting in no adverse effect on the body weight of the mice (Figure 8E). These results demonstrate that functional bacteria's immunological efficacies and optical properties are highly effective in eradicating drug‐resistant aggressive cancers.

Finally, anticancer efficacies of other intratumoral bacteria [*Lactococcus sp*. (i‐LS) and *Enterococcus faecalis* (i‐EF)], isolated from solid tumor biopsies without i.v. injection of ca‐RP, were further investigated using Colon‐26‐bearing mice (Figure S15, Supporting Information). Isolations of i‐LS and i‐EF were successfully assessed using similar means of extractions of AUN, A‐gyo, and UN‐gyo except for using Luria broth (LB) medium (Figure S15A, Supporting Information). Interestingly, the homology of i‐LS was closest to *Lactococcus formosensis* (97.69%) or *Lactococcus garvieae* (97.48%) using Basic Local Alignment Search Tool (BLAST) analyses. In other words, gene identification exhibited that i‐LS is potentially a new strain because the similarity of the 16S rRNA gene of i‐LS is less than 98.7% compared with the data‐based bacterial strains (Table S5, Supporting Information).^[^ ^37^ ^]^ Meanwhile, gene identification confirmed i‐EF as 100% pure EF (Table S6, Supporting Information). In any case, both i‐LS and i‐EF showed tumor suppression effects in Colon‐26‐bearing mice, although slight body weight reductions were observed after their injections (Figure S15B–G, Supporting Information). i‐LS represented stronger anticancer efficacy and achieved 33% CR. These results indicate a high potentiality of various intratumoral microbiota as a powerful antitumor medicine.

After all, the microbial consortium AUN exhibited stronger efficacies than A‐gyo and other control groups (e.g., A‐gyo + UN‐gyo and UN‐gyo) against various types of cancer presumably because of a symbiotic relationship between A‐gyo and UN‐gyo. We believe that naturally isolated AUN composed of A‐gyo and UN‐gyo is an excellent modality for eliminating various tumors, and this natural combination should not be physically separated to keep its innate strong anticancer effect. RP itself was essential to obtain such highly effective anticancer AUN from solid tumor biopsies, although successful isolation of AUN was unexpected at all at the beginning of the work. In any case, we concluded that functional bacteria AUN could serve as a universal immuno‐activatable therapeutic “living” agent in various syngeneic cancer models.

## 3 Conclusion

Earlier studies on the correlations between intratumoral bacteria and cancer have mainly concentrated on studying the role of bacteria in tumor pathogenesis and the contribution of the metabolism of bacteria to the adverse effects of chemotherapy. However, the therapeutic activities of intratumoral bacteria have not been explored. We demonstrated that isolated native intratumoral bacteria and their microbial consortium could modulate various immunological activities to eliminate tumors with or without tumor immunogenicity dramatically. They exhibit high biocompatibility in mice and antitumor effectiveness with excellent tumor selectivity through a single administration. The association of intratumoral bacteria and photosynthetic bacteria can work as a potent therapeutic agent and a beneficial cancer diagnosis probe, indicating highly tumor‐specific strong NIR FL emission and immunological elimination under tissue‐penetrable NIR light.

The number of cancer patients worldwide has drastically increased, resulting in annual spending on anticancer drugs of more than US$ 100 billion, and it is predicted to rise continually.^[^ ^38^ ^]^ However, despite the high demand, the price of advanced anticancer medicine has skyrocketed. Moreover, at the peak of the COVID‐19 pandemic, tens of millions of people globally lost their jobs and health coverage.^[^ ^39^ ^]^ Such unfortunate situations affected groups suffering from cancer and poverty. The functional bacteria discovered in this study can be abundantly proliferated in inexpensive media without any complicated process, indicating that they may resolve the troublesome cost issue of anticancer medicines. Further, these functional bacteria would be a useful “hardware” in which genes and the microbial surface can be genetically and chemically manipulated in combination with emerging genetic and nanoengineering approaches for obtaining higher therapeutic performances and multifunction by modulating metabolic pathways and loading with nanoparticles. The bacterial platform technology, which does not require substantial petrochemicals, may also shed light on global environmental concerns because functional bacteria could be alternative eco‐friendly medicinal materials instead of traditional organic anticancer drugs typically synthesized from fossil fuels with massive carbon dioxide emissions.^[^ ^40^ ^]^

More importantly, this study is the first to draw attention to the potential of many intratumoral bacteria, also called microbial dark matter, as an independent natural active anticancer medicine for future innovative cancer treatment. We envision that tailor‐made intratumoral microbiota derived from individual cancer patients might have unique anticancer efficacy and can be used as personalized medicine. Bacterial cancer therapy is now experiencing a renaissance, and the gold rush of research into various intratumoral bacteria for cancer treatment has just begun.

## 4 Experimental Section

### Bacterial Strains and Growth

The bacterial strains tested in this study were purchased from the National Institute of Technology and Evaluation Biological Resource Center (NBRC) (Chiba, Japan). Commercially available *Rhodopseudomonas palustris* (ca‐RP) (NBRC 16 661) was grown anaerobically in liquid cultures at 26–30 °C in ATCC 543 medium under tungsten lamps. Commercially available *Proteus mirabilis* (ca‐PM) (NBRC 3849 and ATCC 35 659) were anaerobically cultured at 30 °C in NBRC 802 medium. All reagents for bacterial culturing were obtained from Nacalai Tesque (Kyoto, Japan), Tokyo Chemical Industry (Tokyo, Japan), and FUJIFILM Wako Pure Chemical (Osaka, Japan).

### Cell Culture

Murine colon carcinoma (Colon‐26) cells were obtained from the Japanese Collection of Research Bioresources Cell Bank (Tokyo, Japan). Murine skin sarcoma (Meth‐A) and murine melanoma (B16F10) cell lines were purchased from the Cell Resource Center for Biomedical Research, Institute of Development, Aging and Cancer Tohoku University (Sendai, Japan). Drug‐resistant mouse mammary tumor (EMT6/AR1) cell line was obtained from KAC Co., Ltd. (Tokyo, Japan). The Colon‐26, Meth‐A, and B16F10 cell lines were cultured in Roswell Park Memorial Institute 1640 Medium (Gibco, Grand Island, NY) containing 10% fetal bovine serum, 2 mm L‐glutamine, 1 mm sodium pyruvate, gentamycin, and penicillin‐streptomycin (100 IU mL^−1^). EM6/AR1 cell line was cultured in cell growth medium No.104 (KAC Co., Ltd.) containing doxorubicin (1 µg mL^−1^). Cells were maintained at 37 °C in a humidified incubator containing 5% CO_2,_ were cryopreserved in multiple vials, and stored in liquid nitrogen. Cell stocks were regularly revived to avoid the genetic instabilities associated with high passage numbers.

### Isolation of Functional Bacteria From Solid Tumors

The animal experiments were conducted following the protocols approved by the Institutional Animal Care and Use Committee (IACUC) of Japan Advanced Institute of Science and Technology (JAIST) (No. 04‐007). Female BALB/cCrSlc mice (*n* = 8; 4 weeks old; average weight = 15 g) were obtained from Japan SLC (Hamamatsu, Japan). Mice bearing the Colon‐26 cell‐derived tumors were generated by injecting 100 µL of the culture medium/Matrigel (Dow Corning, Corning, NY) mixture (v/v = 1:1) containing 1 × 10^6^ cells into the right side of the backs of the mice. After ≈20 days, when the tumor volumes reached ≈400 mm^3^, the mice were intravenously injected with 200 µL culture medium containing ca‐RP (5 × 10^9^ CFU mL^−1^). After 2 days, the tumors were carefully excised. After homogenizing thoroughly with a homogenizer pestle in 1 mL of PBS solution at 4 °C, the mixture was shaken for 20 min at a speed of 380 rpm min^−1^ at 15 °C. The supernatant was diluted 10 times with PBS, and a sample (100 µL) was inoculated onto an agar plate. After incubation for 3 days under anaerobic conditions and tungsten lamps, the red bacterial colonies derived from RP were formed on plates. Several colonies were picked and cultured anaerobically in ATCC 543 medium at 26–30 °C under tungsten lamps for 3 days. Gray‐colored bacteria solution was subcultured in conventional ATCC 543 medium or ATCC 543 medium without L‐cysteine (Cys). Subcultured red bacterial suspension prepared in ATCC 543 medium without Cys was disseminated onto an ATCC 543 agar plate with 0.1% sodium deoxycholate (SD) and without Cys. After 7 days, red and white colonies were observed on the plate. A single red colony was carefully picked up using an inoculating loop and then cultured in an ATCC 543 medium without Cys for 3 days. A prepared red bacterial solution [isolated *Rhodopseudomonas palustris* (i‐RP) (AUN)] was used for further tests.

After subculturing, obtained gray‐colored bacterial solution prepared using conventional ATCC 543 medium was inoculated onto an ordinary ATCC 543 agar plate containing 0.1% SD. White bacterial colonies were formed on the plate. A single white colony was carefully picked up by an inoculating loop and then anaerobically cultured in conventional ATCC 543 medium at 26–30 °C under tungsten lamps for 3 days. A prepared gray bacterial solution [isolated *Proteus mirabilis* (i‐PM) (A‐gyo)] was used for further tests.

Besides, a single red colony prepared from AUN solution was carefully picked up using a 29‐gauge syringe needle (Myjector Insulin Syringe, 0.337 mm diameter, Terumo, Tokyo, Japan) under stereo microscopy (M2‐1139‐01; AS ONE, Osaka, Japan), and then anaerobically cultured in an ATCC 543 medium without Cys at 26–30 °C under tungsten lamps for 3 days. A prepared red solution [super‐purified RP (sp‐RP) (UN‐gyo)] was used for further tests.

The AUN was identified as a microbial consortium of RP and PM using techniques such as colony assay, optical microscopy, and gene identifications with Basic Local Alignment Search Tool (BLAST) analyses using 16S rRNA primers (listed in Table S7, Supporting Information). The gene identifications were performed by BEX Co. (Tokyo, Japan). The AUN's microbial consortium ratio was measured as RP:PM = 97:3 by fluorescent microscopy and colony assay. The A‐gyo and UN‐gyo were confirmed as pure bacteria by colony assays, optical microscopy, and gene identifications.

*Lactococcus sp*. (i‐LS) and *Enterococcus faecalis* (i‐EF) were also isolated from solid tumors (≈400 mm^3^) in Colon‐26‐bearing mice using a syringe needle and stereomicroscope without i.v. injections of RP, and anaerobically cultured in Luria broth (LB) agar plate and LB medium at 26–30 °C under tungsten lamps. i‐LS was subcultured in Pearl Core E‐MC64 medium (Eiken Chemical, Tokyo, Japan) at 32 °C using an incubator (i‐CUBE FCI‐280HG; AS ONE). i‐EF was subcultured in LB medium (Nacalai Tesque) at 26–30 °C under tungsten lamps. The obtained i‐LS and i‐EF were genetically identified as pure strains by 16S rRNA technique and BLAST analyses similar to the identifications of other bacterial strains in this study.

### Preparation of ICG‒CRE‒A‐gyo

ICG‒CRE‒A‐gyo was prepared as reported previously.^[^ ^32^ ^]^ Briefly, indocyanine green (ICG, 1 mg mL^−1^; Tokyo Chemical Industry) was dissolved in PBS with 5% cremophor EL (Nacalai Tesque) using sonication to obtain ICG‒CRE nanoparticles. The medium containing A‐gyo was centrifuged at 3000 × *g* for 5 min at a temperature of 25 °C and washed with PBS. Following this, the concentration of the bacterial suspension was adjusted to 2 × 10^9^ CFU mL^−1^ and centrifuged to obtain a bacterial pellet. ICG‒CRE (ICG concentration = 1 mg mL^−1^) was added, and the cell suspension was incubated overnight at 37 °C. Further, the samples were centrifuged to remove the unattached dye molecules, and the modified bacteria were resuspended in PBS. The viabilities of ICG‒CRE‒A‐gyo were measured using a bacterial counter (DU‐AA01NP‐H; PHC Co., Ltd., Tokyo, Japan) in addition to an active colony assay.

### Optical Characterizations of Bacteria

Absorption spectra of bacterial solutions were recorded at 20 °C using a UV–vis–NIR spectrophotometer (V‐730 BIO; Jasco, Tokyo, Japan). The fluorescence of bacterial suspensions was measured using a fluorescence spectrometer (FP‐8600 NIR Spectrofluorometer; Jasco, Tokyo, Japan). The amount of loaded and internalized ICG into bacteria was calculated from the collected supernatant of washed i‐PM after modification of ICG‒CRE by a UV–vis–NIR spectrophotometer.

### FL Microscopy Imaging of Bacteria

Bacterial solutions (20 µL, 5 × 10^8^ CFU mL^−1^) were plated on glass coverslips (AGC Techno glass, Shizuoka, Japan) and then observed using a fluorescence microscopy system (IX73) and cellSens V3.1 software (Olympus) equipped with a mirror unit (IRDYE800‐33LP‐A‐U01; Semrock, Lake Forest, IL) and objectives (×60 magnification, aperture 1.35; UPLSAPO60X, Olympus or ×100 magnification, aperture 0.95; PLFLN100X, Olympus) at 20 °C. Besides, the NIR FL images and NIR FL intensity of ICG‒CRE‒A‐gyo were analyzed using a light microscopy system (BZ‐X800, Keyence, Osaka, Japan) and ImageJ software (Fiji). Before analyzing, ICG‒CRE‒A‐gyo was incubated in fresh PBS buffer for 2, 4, 12, and 24 h at 25 and 4 °C, respectively.

### Intracellular Penetration of Bacteria

Colon‐26 cells (2.5 × 10^5^ cells well^−1^) were seeded in poly‐*L*‐lysine coated glass bottom dishes (Matsunami glass, Osaka, Japan) and allowed to adhere overnight. Cells were then exposed to 1 × 10^7^ CFU of AUN or 1 × 10^7^ CFU of ICG‒CRE‒A‐gyo for 4 h at 4 or 37 °C in a fridge or a humidified incubator containing 5% CO_2_. After washing thoroughly with fresh PBS solution, Colon‐26 cells were observed using a fluorescence microscopy system (IX73) equipped with a mirror unit (IRDYE800‐33LP‐A‐U01; Semrock, Lake Forest, IL) and an objective (×20 magnification, aperture 0.75; UPLSAPO20X, Olympus) at room temperature.

### Tumor Spheroids

Colon‐26 cells (1 × 10^4^ cells well^−1^) were seeded in a 3D culture spheroid plate (Cell‐able BP‐96‐R800; Toyo Gosei, Tokyo, Japan) according to the manufacturer's instructions provided with the plate. Cells were cultured for 7 days at 37 °C in a humidified incubator containing 5% CO_2_. The medium was replaced every 2 days. Prepared spheroids were then exposed to 1 × 10^6^ CFU of AUN or 1 × 10^6^ CFU of ICG‒CRE‒A‐gyo for 24 h at 37 °C in a humidified incubator containing 5% CO_2_. After washing thoroughly with fresh PBS solution, spheroids were observed using a fluorescence microscopy system (IX73) equipped with a mirror unit (IRDYE800‐33LP‐A‐U01; Semrock) and an objective (×20 magnification, aperture 0.75; UPLSAPO20X, Olympus) at 20 °C.

### FISH Assay

Microbial FISH assay of AUN and A‐gyo in a tumor slice was assessed using a *Rhodopseudomonas* (Probe name, Rhodopseud; Accession no. pB‐1634; Chromosome Science Labo, Hokkaido, Japan) and PM (Probe name, EUB338; Chromosome Science Labo) specified microbial FISH probes according to the manufacturer's instructions provided with the kit. Rhodopseud and EUB338 Probes were labeled with cyanine 3 (Cy3) and fluorescein isothiocyanate (FITC), respectively. The specificity of the probes was confirmed using the database at ProbeBase (University of Vienna, Vienna, Austria) and the previous literature.^[^ ^41^ ^]^ Briefly, after deparaffinizing the tumor sections and treating them with 0.02% pepsin (FUJIFILM Wako Pure Chemical)/0.1N HCl solution (FUJIFILM Wako Pure Chemical), the sections were stained with Rhodopseud probe (5“‐GACTTAGAAACCCGCCTACG‐3”) (listed in Table S9, Supporting Information) or EUB338 probe (5“‐GCCCCTGCTTTGGTC‐3”) (listed in Table S9, Supporting Information) and DAPI (Dojindo Laboratories) and examined using fluorescent microscopy (IX73).

### In Vivo Anticancer Therapy

The Colon‐26 tumor‐bearing mice (female; about 8 weeks; *n* = 3–10; average weight = 18 g; average tumor size ≈400 mm^3^; BALB/cCrSIc; Japan SLC) were intravenously injected in the tail vein with culture medium (200 µL) containing AUN (5 × 10^9^ or 5 × 10^8^ CFU mL^−1^), A‐gyo (5 × 10^8^ or 1 × 10^7^ CFU mL^−1^), A‐gyo + UN‐gyo (5 × 10^9^ CFU mL^−1^, A‐gyo:UN‐gyo = 3:97), UN‐gyo (5 × 10^9^ CFU mL^−1^), ca‐RP (5 × 10^9^ CFU mL^−1^), or ca‐PM (5 × 10^8^ CFU mL^−1^). The control experiments were also performed using PBS (200 µL). The tumor formation and overall health (viability and body weight) were monitored daily. The tumor volume was calculated using V = L × W^2^/2, where L and W denote the length and width of the tumor, respectively. The survival ratio of Colon‐26 tumor‐bearing mice (*n* = 10 biologically independent mice) was also measured during treatment for 40 days. When the tumor volumes reached more than 2000 mm^3^, the mice were euthanized as the endpoint according to the guidelines of the Institutional Animal Care and Use Committee of JAIST.

For studying cancer immunity, the CR‐achieved mice (Day 120) by bacterial therapy (*n* = 5 biologically independent mice) were injected at the right side of the backs of mice with 100 µL of the culture medium/Matrigel mixture (v/v = 1:1) containing 1 × 10^6^ cells of Colon‐26. After 20 days, no reoccurrences of the tumor were carefully evaluated during 20 days. The control experiments were also performed after i.v. injections of PBS (200 µL) into BALB/c mice (female; about 8 weeks; *n* = 5–10; average weight = 18 g; BALB/cCrSIc; Japan SLC). After 120 days, 100 µL of the culture medium/Matrigel mixture (v/v = 1:1) containing 1 × 10^6^ cells of Colon‐26 was subcutaneously injected into the right side of the backs of mice. The volumes of tumors were measured after 20 days, similar to the aforementioned in vivo anticancer therapy against the Colon‐26 syngeneic mouse model.

For investigating in vivo anticancer therapy using a sarcoma model, mice bearing Meth‐A cell‐derived tumors were generated by injecting 100 µL of the culture medium/Matrigel (Dow Corning, Corning, NY) mixture (v/v = 1:1) containing 1 × 10^6^ cells into the right side of the backs of the mice (female; 5 weeks; *n* = 5; average weight = 18 g; BALB/cCrSIc; Japan SLC). After ≈10 days, when the tumor volumes reached ≈100 mm^3^, the mice were intravenously injected with 200 µL of culture medium containing AUN (5 × 10^9^ CFU mL^−1^) or A‐gyo (5 × 10^8^ CFU mL^−1^). The control experiments were also performed by i.v. injection of PBS (200 µL) into a sarcoma model. The tumor volume, overall health (viability and body weight), and survival ratio of mice were investigated, similar to the anticancer study of Colon‐26 tumor‐bearing mice.

To obtain a metastatic lung cancer‐bearing animal model, female C57BL/6 mice (5 weeks; *n* = 5; average weight = 17 g; C57BL/6NCrSlc; Japan SLC) were injected with 100 mL of 5 × 10^6^ of murine melanoma (B16F10) cells using a 29‐gauge needle through a tail vein (Day 0). Then, 200 µL of culture medium containing AUN (5 × 10^9^ CFU mL^−1^) or A‐gyo (5 × 10^8^ CFU mL^−1^) was intravenously injected after B16F10 cell implantation for 7 days. Animals were sacrificed on day 14, and their lungs were harvested. The lung weight of each mouse treated with PBS or bacteria was measured on the same date. The control experiments without any treatments (inoculations of melanoma and bacteria) were also performed.

To investigate the efficacy of bacteria against drug‐resistant cancer model, 100 µL of the culture medium/Matrigel (Dow Corning, Corning, NY) mixture (v/v = 1:1) containing 1 × 10^6^ cells of EMT6/AR1 were subcutaneously inoculated into the right side of the backs of the mice (female; 5 weeks; *n* = 5; average weight = 18 g; BALB/cCrSIc; Japan SLC). After ≈10 days, when the tumor volumes reached ≈200 mm^3^, the mice were intravenously injected with 200 µL culture medium containing AUN (10 × 10^9^ CFU mL^−1^) or A‐gyo (5 × 10^8^ CFU mL^−1^). The control experiments were also performed using PBS (200 µL). The tumor volume, overall health (viability and body weight), and survival ratio of mice were also studied.

For in vivo tumor tests using i‐LS and i‐EF, the Colon‐26 tumor‐bearing mice (female; about 8 weeks; *n* = 3; average weight = 17 g; average tumor size ≈100 mm^3^; BALB/cCrSIc; Japan SLC) were intravenously injected in the tail vein with culture medium (200 µL) containing i‐LS (5 × 10^9^ CFU mL^−1^) or i‐EF (1 × 10^9^ CFU mL^−1^). The control experiments were performed using PBS (200 µL). The tumor volume, overall health (viability and body weight), and the survival ratio of mice were investigated.

### Biodistribution of Bacteria in a Tumor Model

The Colon‐26 tumor‐bearing mice (female; about 8 weeks; *n* = 3; average weight = 18 g; average tumor size ≈400 mm^3^; BALB/cCrSIc; Japan SLC) were intravenously injected in the tail vein with culture medium (200 µL) containing AUN (5 × 10^9^ CFU mL^−1^) or A‐gyo (5 × 10^8^ CFU mL^−1^). The control experiments were performed using PBS (200 µL). After 25 days, the organs were carefully excised and weighed. After homogenizing thoroughly with a pestle in 1 mL of PBS solution at 4 °C, the mixture was shaken for 20 min at a 380 rpm min^−1^ speed at 15 °C. The supernatant was diluted 10 times with PBS, and a sample (100 µL) was then inoculated onto an agar plate. After incubation under anaerobic conditions for 7 days, the formed bacterial colonies were carefully established. For counting bacterial colonies, the supernatant was diluted 0, 10, 100, and 1000 times with PBS, and then a sample (5 µL) was inoculated onto an agar plate as mentioned above. Finally, formed bacterial colonies were manually counted.

### In Vivo Fluorescent Bio‐Imaging

To monitor the NIR FL intensity due to the tumor‐targeting effect of *R. palustris* (ca‐RP, UN‐gyo, and AUN) in mice, Colon‐26 tumor‐bearing mice (female; about 8 weeks; *n* = 4; average weight = 18 g; average tumor size = 400 mm^3^; BALB/cCrSIc; Japan SLC) were injected intravenously with culture medium containing *R. palustris* (200 µL, 1 × 10^9^ CFU). The whole body of mice was imaged using an in vivo FL imaging system (VISQUE InVivo Smart‐LF, Vieworks, Anyang, Republic of Korea) with a 3 s exposure time and indocyanine green (ICG) filter (*E*_x_, 740–790 nm; *E*_m_, 810–860 nm) 5 days postinjection. The FL images were acquired and analyzed using CleVue software.

### Immunohistochemistry Staining of Tumor Tissues

The Colon‐26 tumor‐bearing mice (female; about 8 weeks; *n* = 4; average weight = 18 g; average tumor size = 400 mm^3^; BALB/cCrSIc; Japan SLC) were euthanized the next day after administration of PBS (200 µL)/ca‐RP (200 µL, 1 × 10^9^ CFU)/UN‐gyo (200 µL, 1 × 10^9^ CFU)/A‐gyo + UN‐gyo (200 µL, 1 × 10^9^ CFU, A‐gyo:UN‐gyo = 3:97)/A‐gyo (200 µL, 1 × 10^8^ CFU)/AUN (200 µL, 1 × 10^9^ CFU) injection intravenously. Then, the tumor tissues from the different treatment groups were harvested on day 1 for IHC staining. Analysis was performed by Biopathology Institute Co., Ltd. (Oita, Japan) using standard protocols. Briefly, primary tumors were surgically removed, fixed in 10% formalin, processed for paraffin embedding, and cut into 3–4 µm thick sections. After incubation with primary antibodies (listed in Table S8, Supporting Information), the sections were stained with hematoxylin and examined using light microscopy (IX73). The areas showing positive staining in tumor tissues were analyzed using a light microscopy system (BZ‐X800) and hybrid cell count and microcell count software (Keyence).

### Flow Cytometry

The Colon‐26 tumor‐bearing mice (female; about 8 weeks; *n* = 3; average weight = 18 g; average tumor size = 400 mm^3^; BALB/cCrSIc; Japan SLC) were euthanized the next day after administration of PBS (200 µL)/A‐gyo (200 µL, 1 × 10^8^ CFU)/AUN (200 µL, 1 × 10^9^ CFU) injection intravenously. To analyze the immune cells in tumors, tumors were collected from mice in different groups after i.v. injection of sample for 3 or 24 h, homogenized into single‐cell suspensions with Accumax (Nacalai Tesque), and 1 × 10^6^ cells were stained with fluorescein isothiocyanate (FITC)‐ or Alexa Fluor 488‐labeled antibodies (listed in Table S8, Supporting Information) according to the manufacturer's protocols, and then classified by flow cytometry (CyFlow Cube 6, Sysmex, Kobe, Japan) by analyzing 4 × 10^4^ cells for each sample.

### Blood Tests

The CBC and biochemical parameters were investigated by Japan SLC and Oriental Yeast Co. (Tokyo, Japan). BALB/cCrSlc mice (female; 10 weeks; *n* = 5; average weight = 21 g; Japan SLC) were injected in the tail vein with culture medium containing bacteria (200 µL, 1 × 10^6^ CFU) or PBS (200 µL). The blood samples were collected from the inferior vena cava of the mouse after 30 days.

### Statistical Analysis

All experiments except those for the Supporting Information were performed in triplicates and repeated three or more times. Quantitative values are expressed as the mean ± standard error of the mean (SEM) of at least three independent experiments. Statistical differences were identified by the Student's one‐sided/two‐sided *t*‐test, two‐way analysis of variance (ANOVA), or Log‐rank (Mantel‐Cox) test using GraphPad Prism, version 9.4.0 (GraphPad Software, Boston, MA). A P value of less than 0.05 was considered statistically significant.

## Conflict of Interest

EM is inventor of the pending patents that cover a part of key ideas of intratumoral oncolytic bacteria (JP2022/08 2458 and JP2022/08 2460, applied by Japan Advanced Institute of Science and Technology). The authors declare no other competing interests.

## Supporting information

Supporting Information

## Data Availability

The data that support the findings of this study are available from the corresponding author upon reasonable request.
