# Supplementary material for: Discovery of Intratumoral Oncolytic Bacteria Toward Targeted Anticancer Theranostics
Source: Adv Sci (Weinh). 2023 May 7;10(20):2301679. doi: 10.1002/advs.202301679 (PMC10369285; doi:10.1002/advs.202301679)
Supplement: Supplementary file 1 — Supporting Information [file ADVS-10-2301679-s001.pdf]

ADVANCED SCIENCE
Open Access

Supporting Information

for *Adv. Sci.*, DOI 10.1002/advs.202301679

Discovery of Intratumoral Oncolytic Bacteria Toward Targeted Anticancer Theranostics

*Yamato Goto, Seigo Iwata, Mikako Miyahara and Eijiro Miyako**

Supporting Information

Discovery of Intratumoral Oncolytic Bacteria Toward Targeted Anticancer Theranostics

*Yamato Goto, Seigo Iwata, Mikako Miyahara, and Eijiro Miyako**

Graduate School of Advanced Science and Technology, Japan Advanced Institute of Science and Technology, 1-1 Asahidai, Nomi, Ishikawa 923-1292, Japan

*Corresponding author: E-mail: e-miyako@jaist.ac.jp

Supporting Figures & Tables

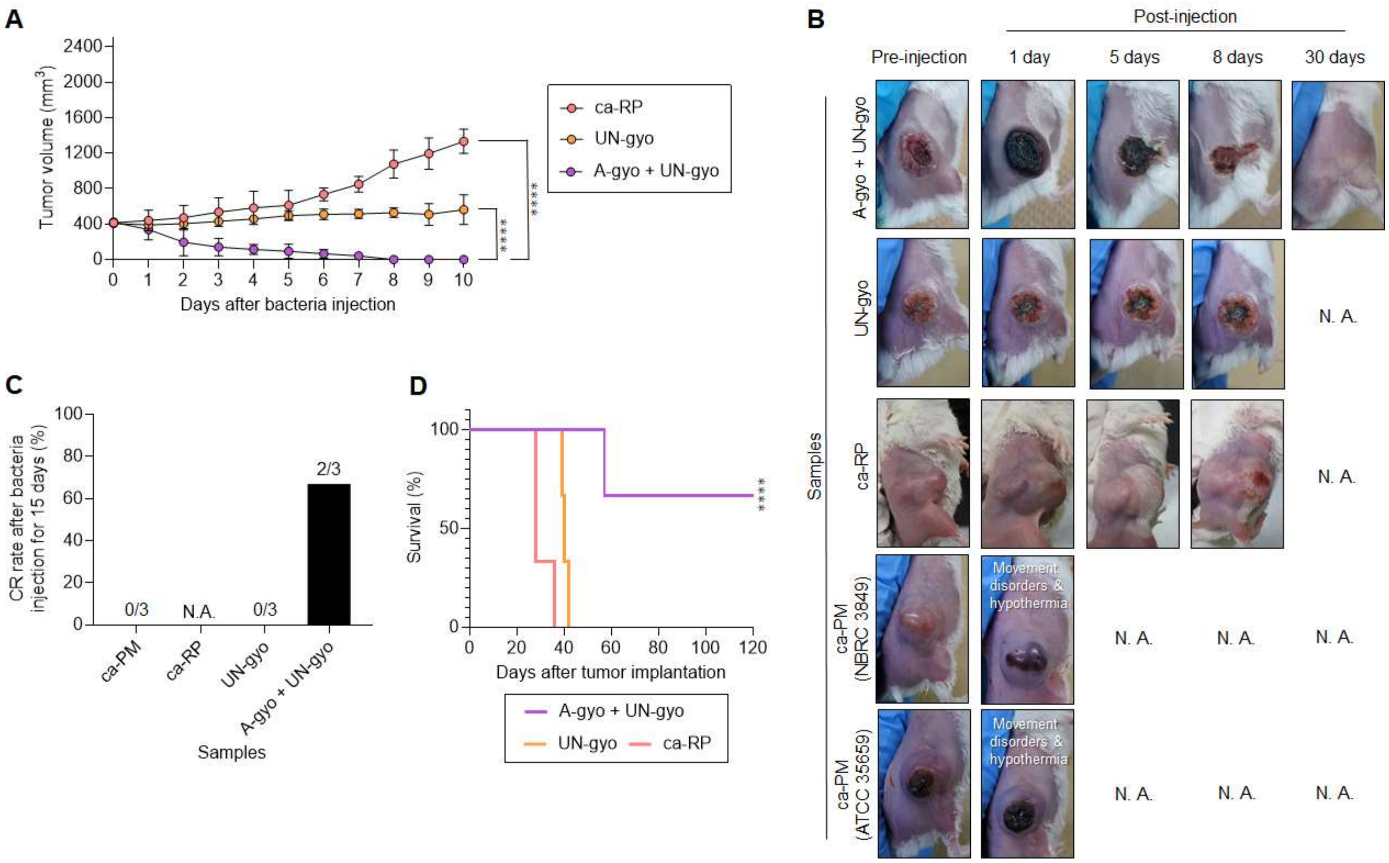

Figure S1. Control bacteria-based cancer treatment for Colon-26-tumor-bearing mice. A) *In vivo* anticancer effect of functional bacteria. The suspension of bacteria was intravenously injected into Colon-26-bearing mice. Data are represented as mean ± standard errors of the mean (SEM); n = 3 biologically independent mice. ****, p < 0.0001, by two-way ANOVA test. B) Images of mice after each treatment. N. A., not available. C) Complete response (CR) rate of Colon-26-tumor-bearing mice (n = 5 biologically independent mice) at day 15 after bacteria or PBS injection. D) Kaplan–Meier survival curves of Colon-26-tumor-bearing mice (n = 5 biologically independent mice) after tumor implantation. Statistical significance was calculated by comparison with the PBS group. ****, *p* < 0.0001, Log-rank (Mantel-Cox) test.

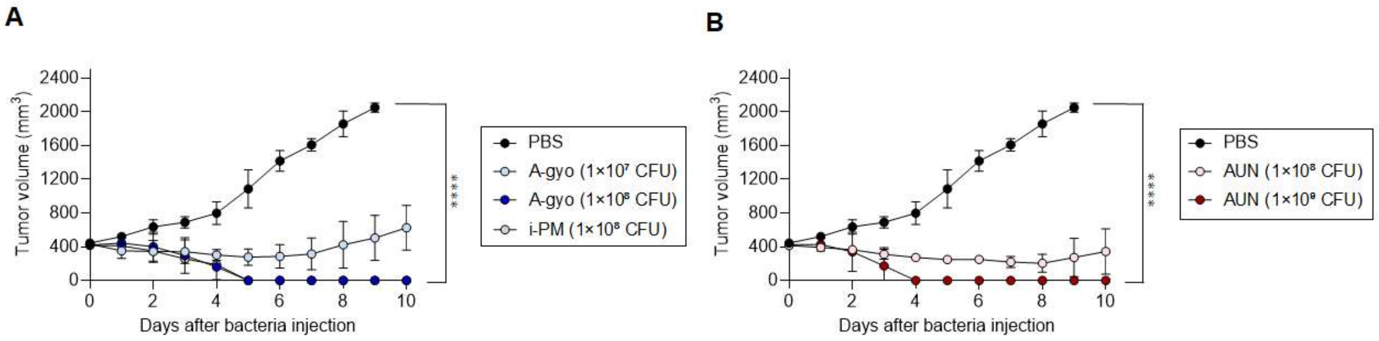

Figure S2. Effect of bacterial concentrations on *in vivo* antitumor efficacy. A) *In vivo* Colon-26 anticancer effect of A-gyo at different concentrations (1×10^7 CFU and 1×10^8 CFU) and i-PM isolated from solid tumor biopsies without i.v. injection of ca-RP (1×10^8 CFU). Data are represented as mean ± standard errors of the mean (SEM); n = 3 (for 1×10^7 CFU) or n = 5 (for 1×10^8 CFU and PBS) biologically independent mice. ****, $p < 0.0001$, by two-way ANOVA test. B) *In vivo* Colon-26 anticancer effect of AUN at different concentrations (1×10^8 CFU and 1×10^9 CFU). Data are represented as mean ± SEM; n = 3 (for 1×10^8 CFU) or n = 5 (for 1×10^9 CFU and PBS) biologically independent mice. ****, $p < 0.0001$, by two-way ANOVA test.

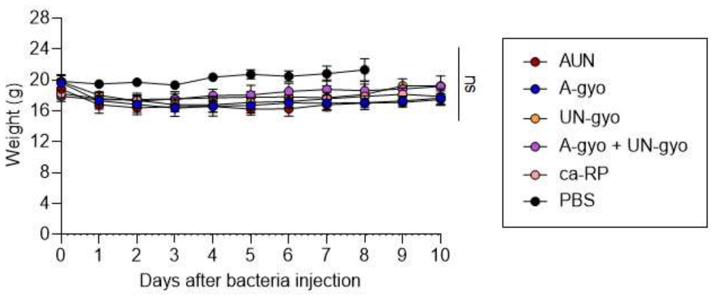

Figure S3. Weight measured after various treatments in Colon-26-tumor-bearing mice. Data are represented as mean ± SEM; n = 5 (AUN, A-gyo, and PBS) or n = 3 (A-gyo + UN-gyo, UN-gyo, and ca-RP) independent experiments. ns, not significant, by two-way ANOVA test.

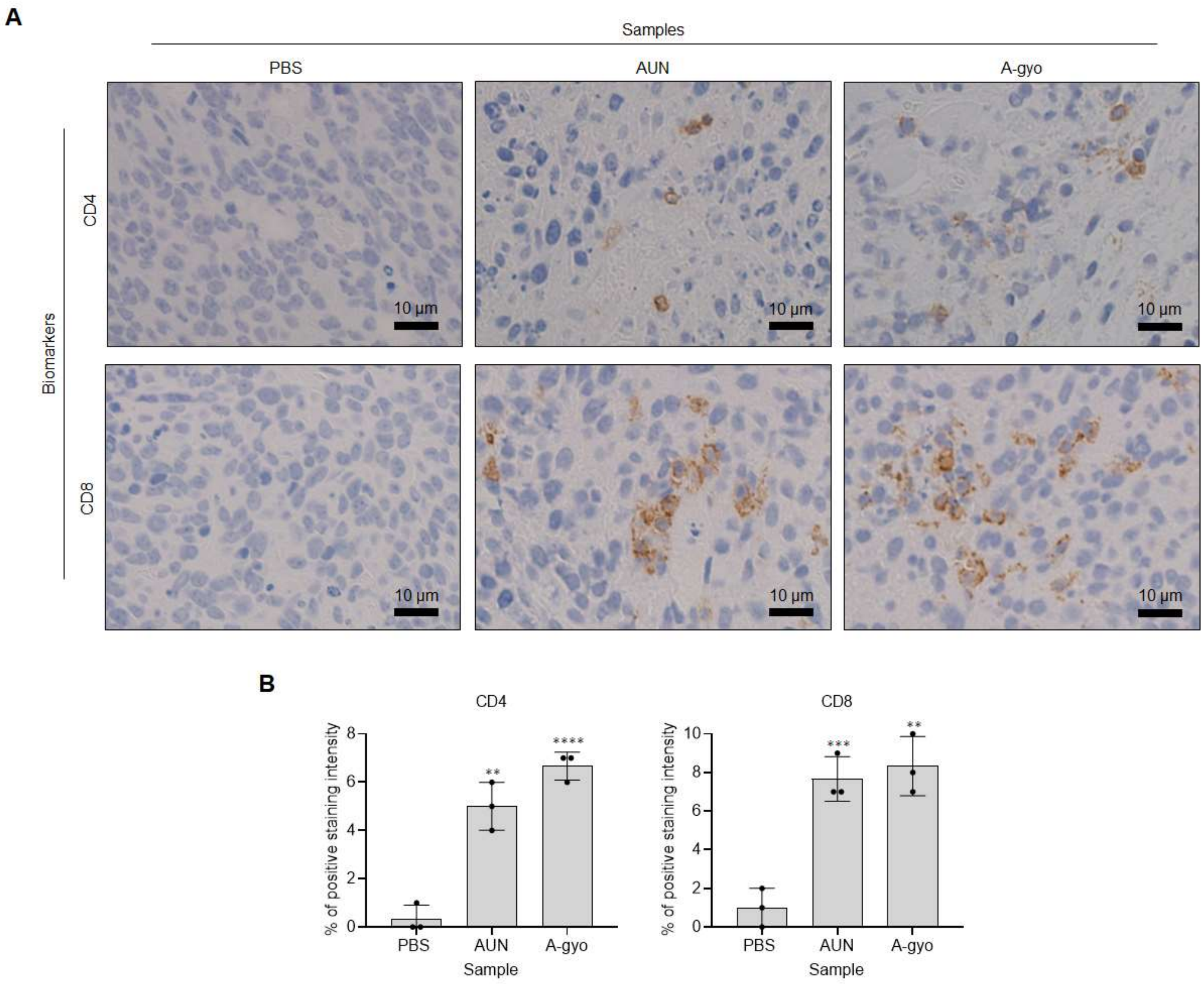

Figure S4. A) IHC (CD4 and CD8) stained tumor tissues collected from mice on day 1 after treatment with PBS, AUN, and A-gyo. B) Statistical analyses of IHC ($CD4^+$ and $CD8^+$ memory T cells). Data are represented as mean ± SEM; n = 3 independent areas (region of interest) in each tumor tissue collected from the groups of mice on day 1 after treatments with A-gyo, AUN, and PBS. Statistical significance was calculated in comparison with the PBS group. **, $p < 0.01$, ***, $p < 0.001$, and ****, $p < 0.0001$, by Student's t one-sided test.

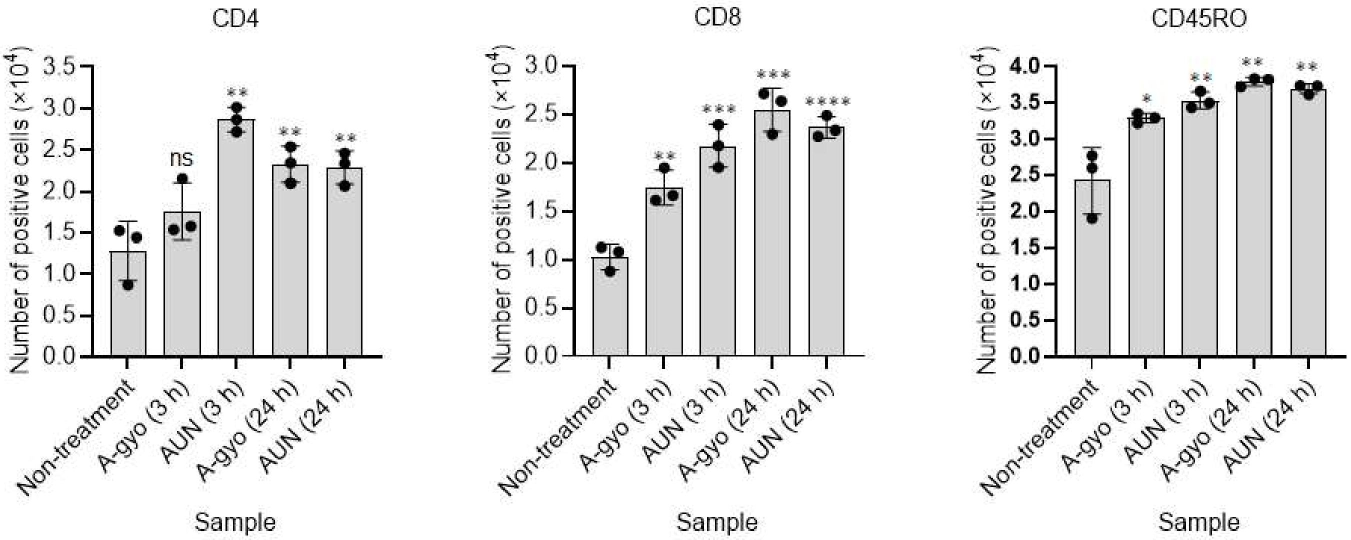

Figure S5. Flow cytometry analyses of expression of CD4$^+$, CD8$^+$, and CD45RO$^+$ memory T cells in tumors after i.v. injection of each sample for 3 and 24 h. Data are represented as mean ± SEM; n = 3 independent tumor tissues. Statistical significance was calculated in comparison with the non-treatment group. ns, not significant, *, $p < 0.05$, **, $p < 0.01$, ***, $p < 0.001$, and ****, $p < 0.0001$, by Student's *t* one-sided test.

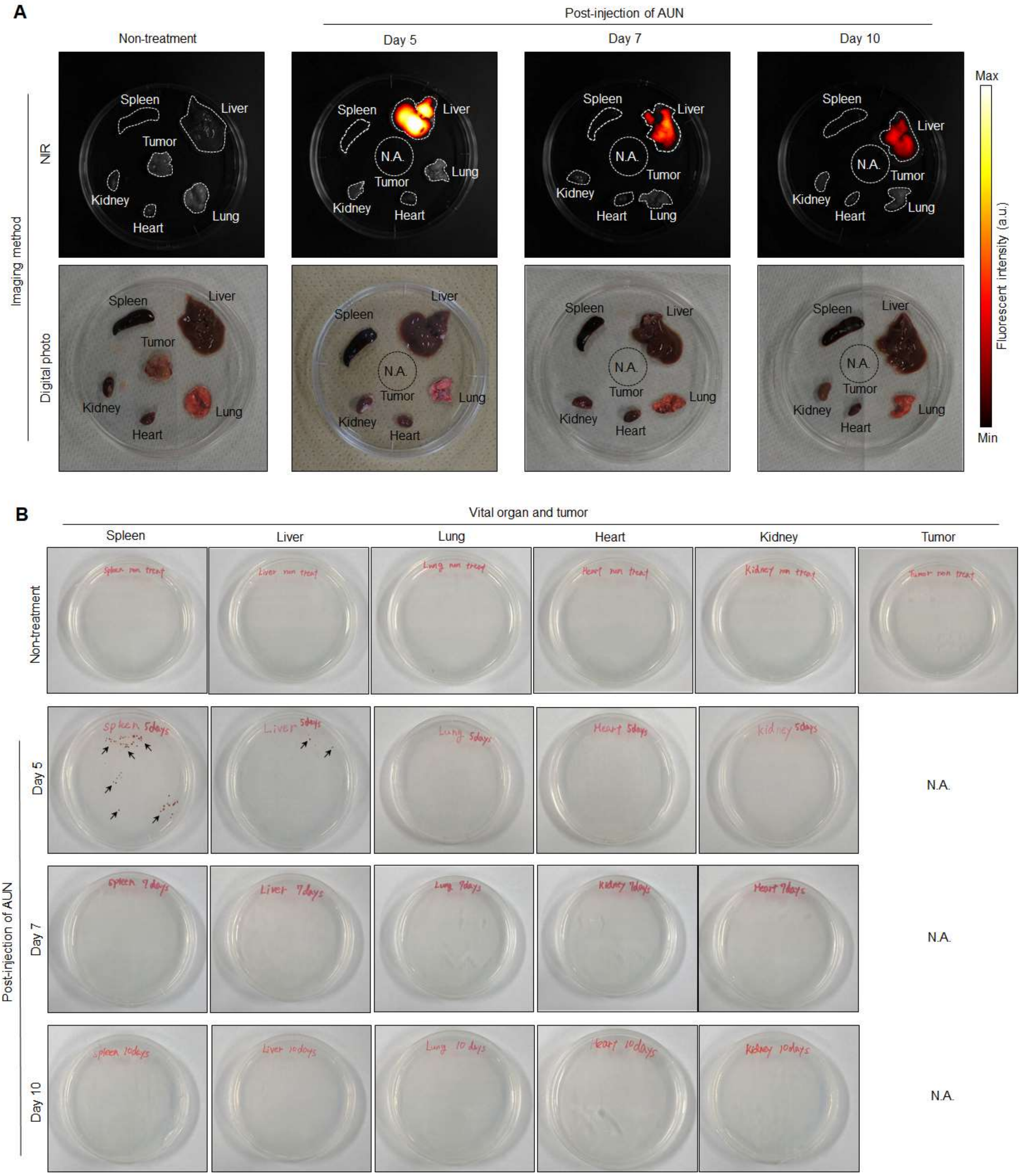

Figure S6. A) *Ex vivo* NIR fluorescent bioimaging of vital organs of Colon26 tumor-bearing mice after i.v. injection of AUN at Day 5, Day 7, and Day 10. N.A.: Tumors were completly disappeared by bacterial treatment. B) Images of bacterial colony of AUN from extracted organs of Colon26 tumor-bearing mice after i.v. injection of AUN at Day 5, Day 7, and Day 10. Black arrows represent bacterial colonies of AUN.

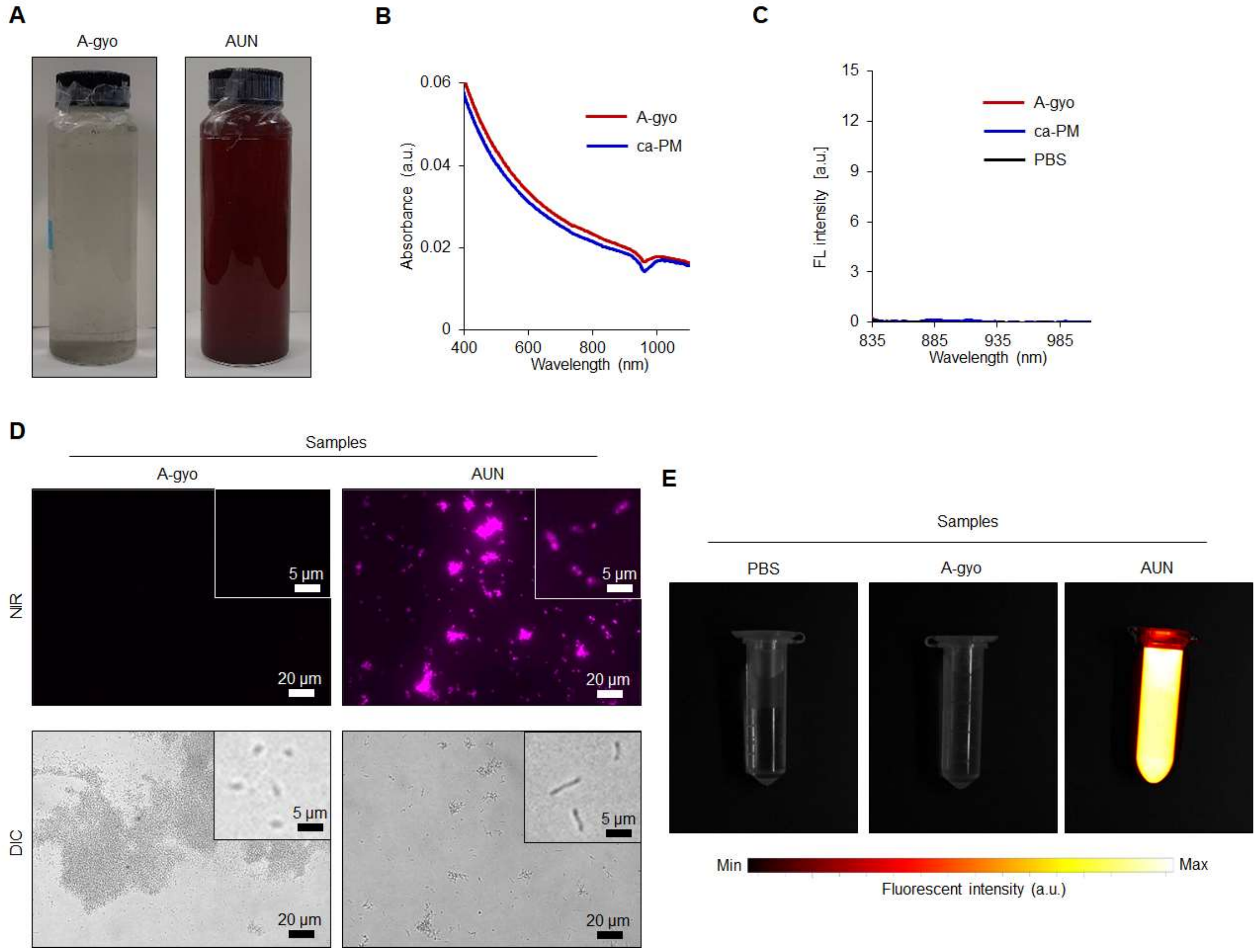

Figure S7. Optical properties of functional bacteria. A) Images of A-gyo (left) and AUN (right) dispersions. B) UV–Vis–NIR absorbance spectra of i-PM and ca-PM. C) Fluorescent emission spectra of A-gyo, ca-PM, and PBS excited at 805 nm. D) *In vitro* NIR fluorescent and differential interference contrast (DIC) imaging of A-gyo and AUN. The bacterial concentration is 5×10^8 CFU mL^{-1}. Upper-right inset of fluorescent/DIC images is the magnified A-gyo and AUN. The bacteria display a pink fluorescence. (**E**) NIR FL images of PBS, A-gyo, and AUN dispersions.

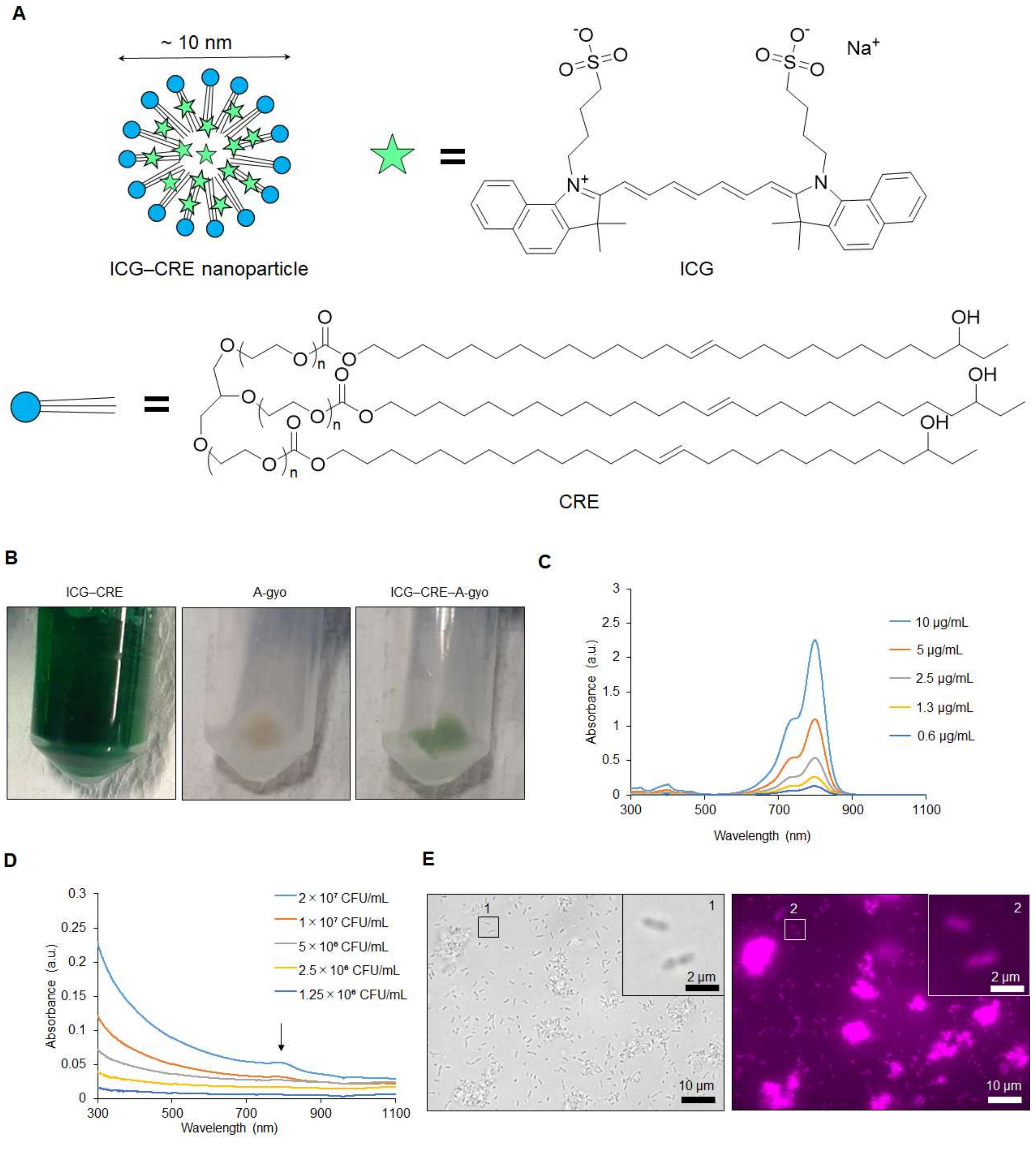

Figure S8. Optical properties of ICG–CRE–A-gyo. A) Schematic illustration of ICG–CRE nanoparticles. B) Images of ICG–CRE dispersion (left), A-gyo (middle), and ICG–CRE–A-gyo (right) pellets. C) UV–Vis–NIR absorbance of ICG–CRE dispersions at different concertation. D) UV–vis–NIR absorbance spectra of ICG–CRE–A-gyo dispersions at different concentrations. The black arrow displays a characteristic peak from an ICG molecule around 800 nm. E) DIC and NIR fluorescent imaging of the prepared ICG–CRE–A-gyo. The numbers (1 and 2) represent the location for magnified images of bacteria.

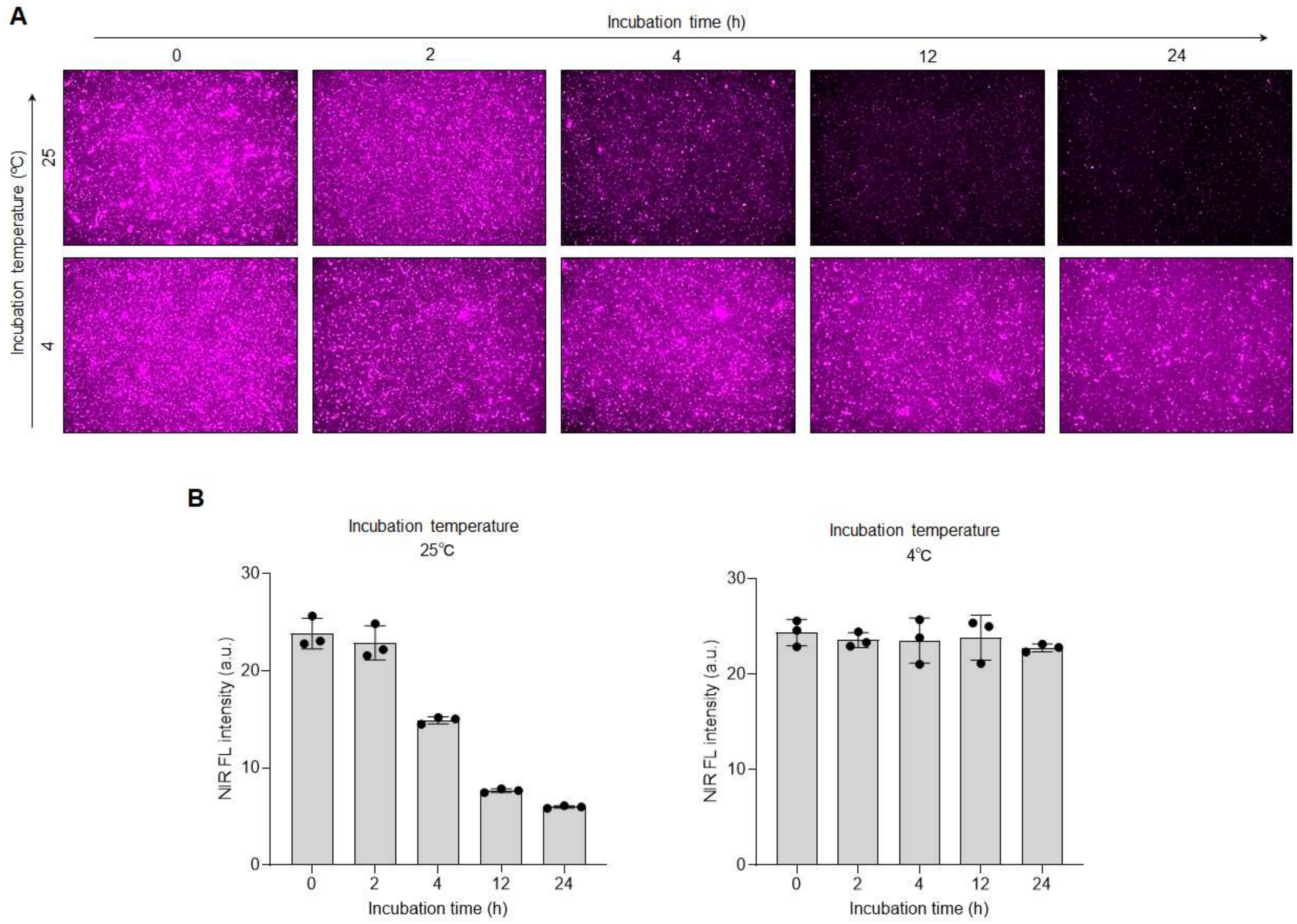

Figure S9. A) NIR FL images and B) NIR FL intensity of ICG–CRE–A-gyo after incubation in PBS buffer for 2, 4, 12, and 24 h at 25 °C and 4 °C, respectively. Bacterial concentration is 2.7 × 10^8 CFU mL^{-1}. Data are represented as mean ± SEM; n = 3 independent areas (region of interest) in each bacterial solutuion after incubation for 2, 4, 12, and 24 h at 25 °C and 4 °C, respectively.

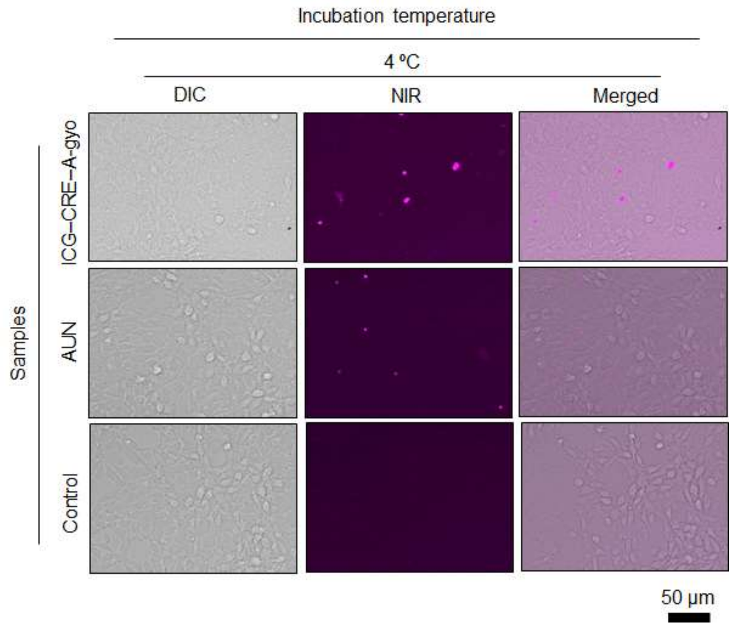

Figure S10. FL images of live Colon-26 cells after treatment with ICG–CRE–A-gyo and AUN for 4 h at 4 °C. The bacteria display a pink fluorescence.

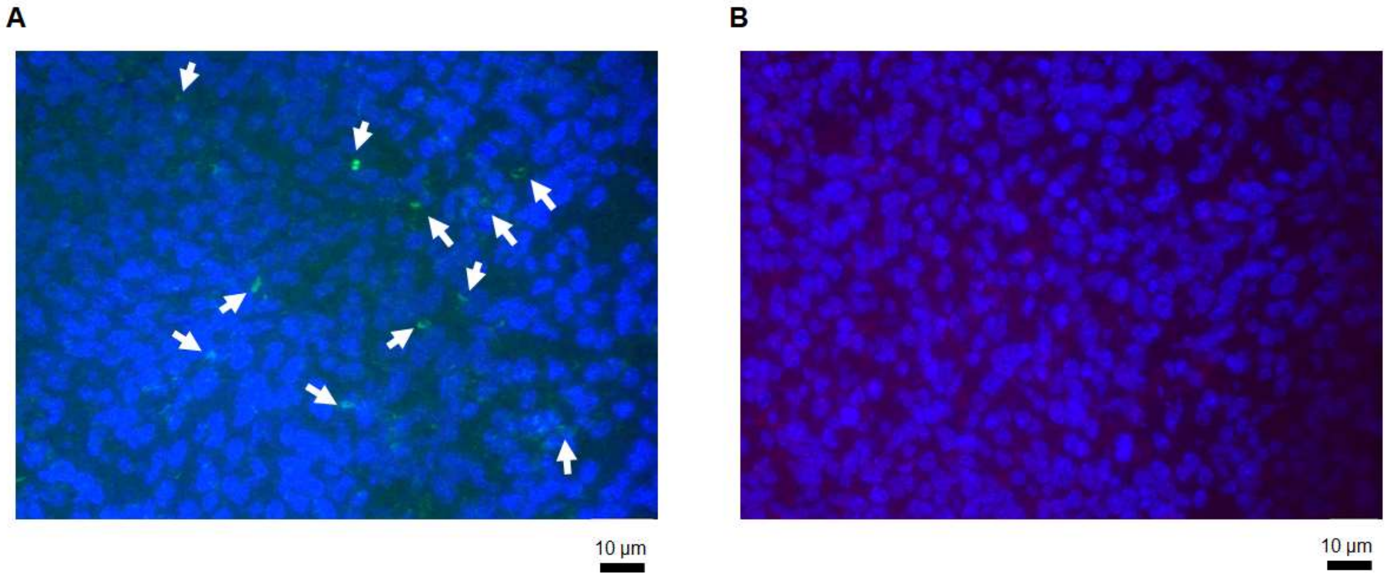

Figure S11. Observation of bacterial distribution in solid tumor tissue using FISH analysis. Bacterial cells and colonies of A) PM and B) RP are colored green and red, respectively. Cancer cells (blue) were counterstained with 4',6-diamidino-2-phenylindole (DAPI). White arrows represent tumor-resident PM colonies.

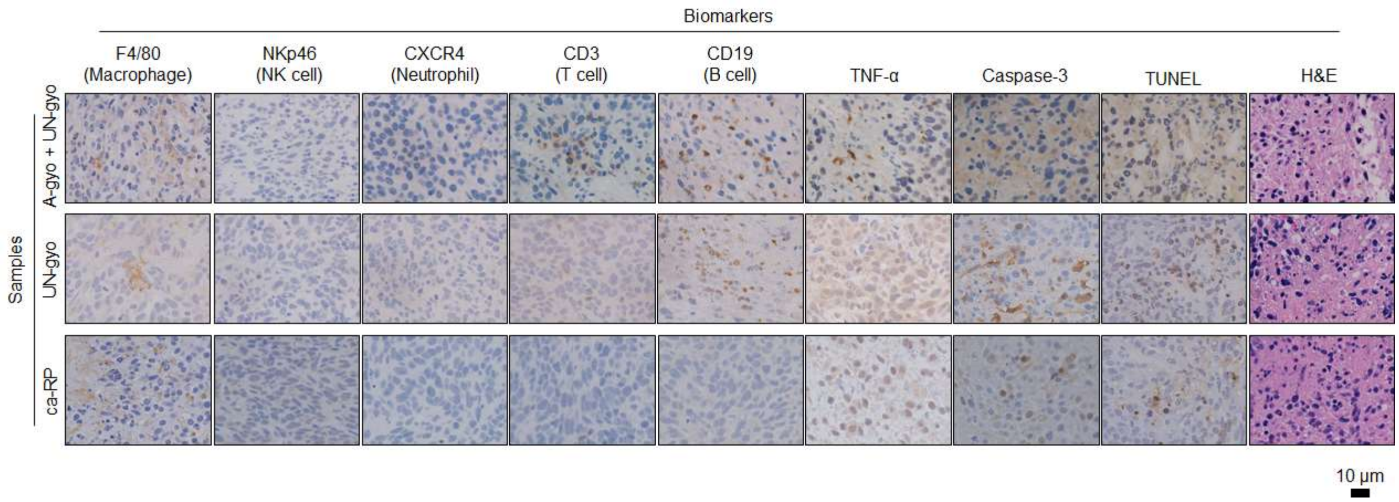

Figure S12. IHC (F4/80, NKp46, CXCR4, CD3, CD19, TNF-α, and caspase-3), TUNEL, and H&E stained tumor tissues collected from the groups of mice on day 1 after treatments with ca-RP, UN-gyo, and A-gyo + UN-gyo.

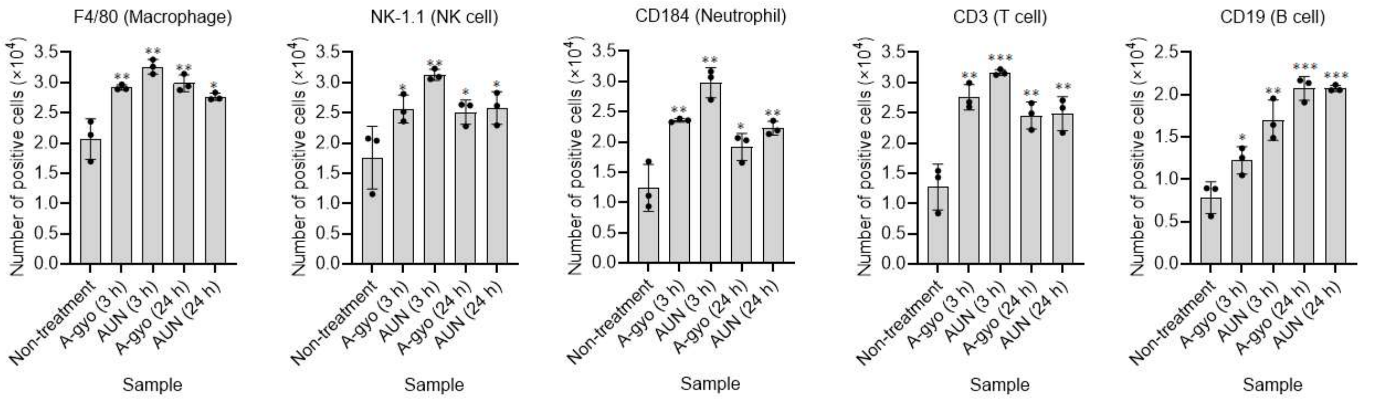

Figure S13. Flow cytometry analyses of expression of various immune cells in tumors at different time points (3 and 24 h) after i.v. injection of each sample. Data are represented as mean ± SEM; n = 3 independent tumor tissues. Statistical significance was calculated in comparison with the non-treatment group. *, $p < 0.05$, **, $p < 0.01$, and ***, $p < 0.001$, by Student's t one-sided test.

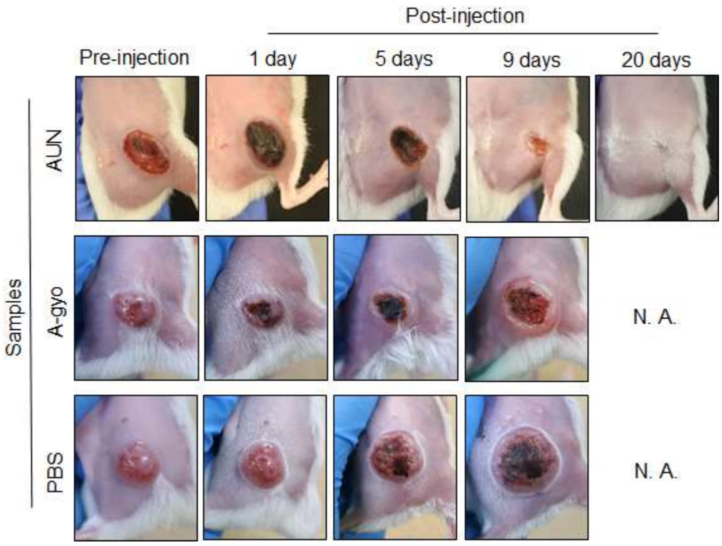

Figure S14. *In vivo* antitumor efficacy of functional bacteria against drug-resistant cancer model. Images of lungs after each treatment.

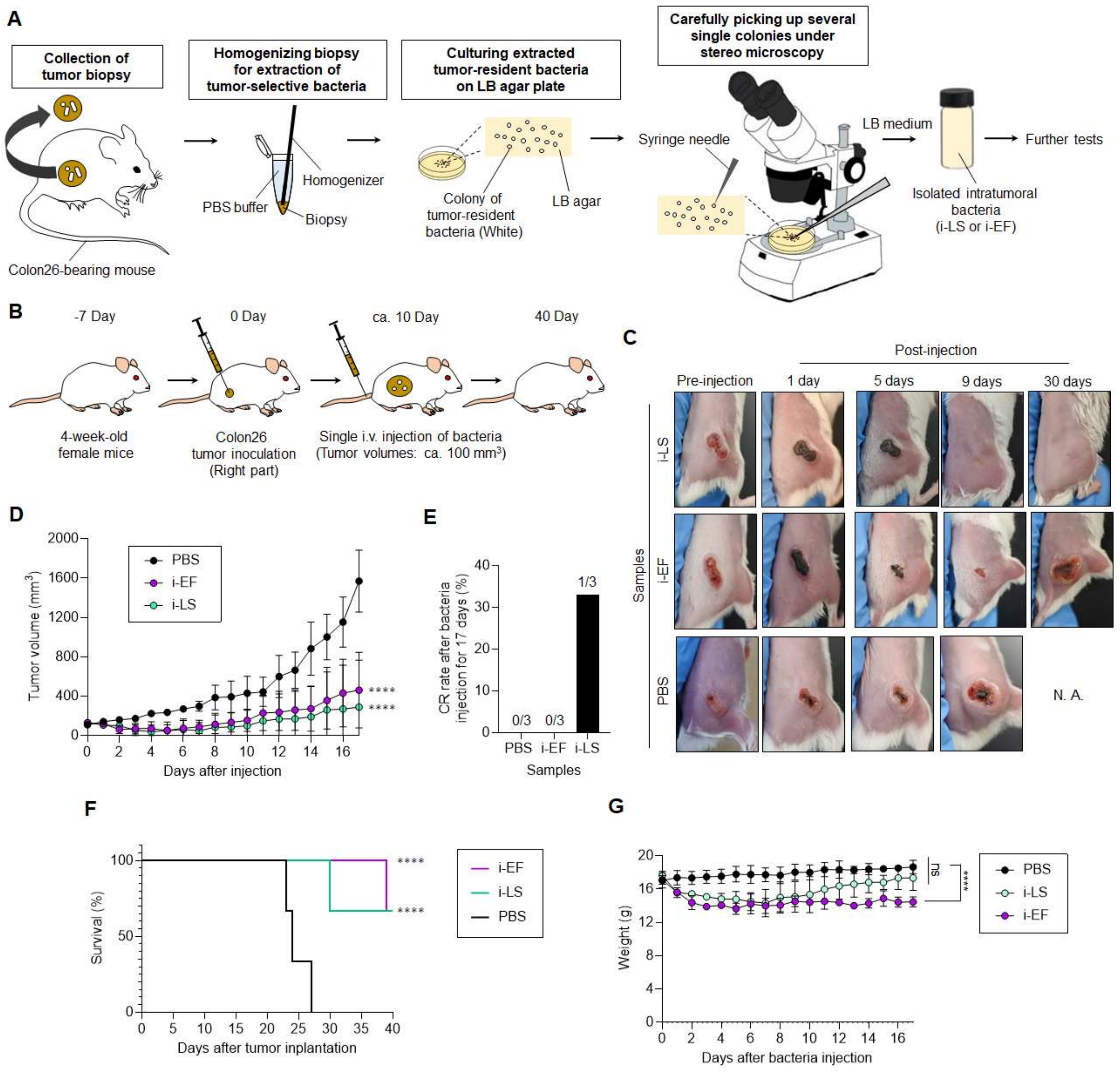

Figure S15. *In vivo* antitumor efficacy of various intramural bacteria. A) Schematic illustration of isolations of i-LS and i-EF from solid tumors. B) Schematic illustration of *in vivo* Colon-26 carcinoma antitumor tests using i-LS and i-EF. C) Images of mice after each treatment. D) *In vivo* anticancer effect of i-LS and i-EF. The PBS or bacterial suspension was intravenously injected into Colon26-bearing mice. Data are represented as mean ± SEM; n = 3 biologically independent mice. Statistical significance was calculated in comparison with the PBS group. ****, $p < 0.0001$. E) The CR rate of Colon-26-bearing mice (n = 3 biologically independent mice) after the injection of bacteria or PBS for 17 days. F) Kaplan–Meier survival curves of Colon26-bearing mice (n = 3 biologically independent mice) after tumor implantation for 40 days. Statistical significance was calculated in comparison with the PBS group. ****, $p < 0.0001$, Log-rank (Mantel-Cox) test. G) Weight of mice after each treatment. Data are represented as mean ± SEM; n = 3 independent experiments. ns, not significant. ****, $p < 0.0001$, by two-way ANOVA test.

Table S1. Obtained 16S rRNA gene sequences of A-gyo.

<Partial gene sequence (758 bp)>

GATTGAACGCTGGCGGCAGGCCTAACACATGCAAGTCGAGCGGTAACAGGrGAAAGCT
TGCTTTCTTGCTGACGAGCGGCGGACGGGTGAGTAATGTATGGGGATCTGCCCGATAG
AGGGGGATAACTACTGGAAACGGTGGCTAATACCGCATAATGTCTACGGACCAAAGCA
GGGGCTCTTCGGACCTTGCACTATCGGATGAACCCATATGGGATTAGCTAGTAGGTGG
GGTAAAGGCTCACCTAGGCGACGATCTCTAGCTGGTCTGAGAGGATGATCAGCCACAC
TGGGACTGAGACACGGCCCAGACTCCTACGGGAGGCAGCAGTGGGGAATATTGCACA
ATGGGCGCAAGCCTGATGCAGCCATGCCGCGTGTATGAAGAAGGCCTTAGGGTTGTAA
AGTACTTTCAGCGGGGAGGAAGGTGATAAGGTTAATACCCTTrTCAATTGACGTTACCC
GCAGAAGAAGCACCGGCTAACTCCGTGCCAGCAGCCGCGGTAATACGGAGGGTGCAA
GCGTTAATCGGAATTACTGGGCGTAAAGCGCACGCAGGCGGTCAATTAAGTCAGATGT
GAAAGCCCCGAGCTTAACTTGGGAATTGCATCTGAAACTGGTTGGCTAGAGTCTTGTA
GAGGGGGGTAGAATTCCATGTGTAGCGGTGAAATGCGTAGAGATGTGGAGGAATACC
GGTGGCGAAGGCGGCCCCCTGGACAAAGACTGACGCTCAGGTGCGAAAGCGTGGGGA
GCAAACA

<Complete gene sequence (1,466 bp)>

ATTGAACGCTGGCGGCAGGCCTAACACATGCAAGTCGAGCGGTAACAGGrAGAAAGCT
TGCTTTCTTGCTGACGAGCGGCGGACGGGTGAGTAATGTATGGGGATCTGCCCGATAG
AGGGGGATAACTACTGGAAACGGTGGCTAATACCGCATAATGTCTACGGACCAAAGCA
GGGGCTCTTCGGACCTTGCACTATCGGATGAACCCATATGGGATTAGCTAGTAGGTGG
GGTAAAGGCTCACCTAGGCGACGATCTCTAGCTGGTCTGAGAGGATGATCAGCCACAC
TGGGACTGAGACACGGCCCAGACTCCTACGGGAGGCAGCAGTGGGGAATATTGCACA
ATGGGCGCAAGCCTGATGCAGCCATGCCGCGTGTATGAAGAAGGCCTTAGGGTTGTAA
AGTACTTTCAGCGGGGAGGAAGGTGATAAGGTTAATACCCTTrTCAATTGACGTTACCC
GCAGAAGAAGCACCGGCTAACTCCGTGCCAGCAGCCGCGGTAATACGGAGGGTGCAA
GCGTTAATCGGAATTACTGGGCGTAAAGCGCACGCAGGCGGTCAATTAAGTCAGATGT
GAAAGCCCCGAGCTTAACTTGGGAATTGCATCTGAAACTGGTTGGCTAGAGTCTTGTA
GAGGGGGGTAGAATTCCATGTGTAGCGGTGAAATGCGTAGAGATGTGGAGGAATACC
GGTGGCGAAGGCGGCCCCCTGGACAAAGACTGACGCTCAGGTGCGAAAGCGTGGGGA
GCAAACAGGATTAGATACCCTGGTAGTCCACGCTGTAAACGATGTCGATTTAGAGGTT
GTGGTCTTGAACCGTGGCTTCTGGAGCTAACGCGTTAAATCGACCGCCTGGGGAGTAC
GGCCGCAAGGTTAAAACTCAAATGAATTGACGGGGGCCCGCACAAGCGGTGGAGCAT
GTGGTTTAATTCGATGCAACGCGAAGAACCTTACCTACTCTTGACATCCAGCGAATCCT
TTAGAGATAGAGGAGTGCCTTCGGGAACGCTGAGACAGGTGCTGCATGGCTGTCGTCA
GCTCGTGTTGTGAAATGTTGGGTTAAGTCCCGCAACGAGCGCAACCCTTATCCTTTGTT
GCCAGCACGTrATGGTGGGAACTCAAAGGAGACTGCCGGTGATAAACCGGAGGAAGGT
GGGGATGACGTCAAGTCATCATGGCCCTTACGAGTAGGGCTACACACGTGCTACAATG
GCAGATACAAAGAGAAGCGACCTCGCGAGAGCAAGCGGAACTCATAAAGTCTGTCGT
AGTCCGGATTGGAGTCTGCAACTCGACTCCATGAAGTCGGAATCGCTAGTAATCGTAG
ATCAGAATGCTACGGTGAATACGTTCCCGGGCCTTGTACACACCGCCCGTCACACCATG
GGAGTGGGTTGCAAAAGAAGTAGGTAGCTTAACCTTCGGGAGGGCGCTTACCACTTTG
TGATTCATGACTGGGGTG

Table S2. Obtained 16S rRNA gene sequences of UN-gyo.

<Partial gene sequence (703 bp)><br><br>GAGCGAACGCTGGCGGCAGGCTTAACACATGCAAGTCGAACGGGCGTAGCAATACGTC<br>AGTGGCAGACGGGTGAGTAACGCGTGGGAACGTACCTTTTGGTTCGGAACAACACAGG<br>GAAACTTGTGCTAATACCGGATAAGCCCTTACGGGGAAAGATTTATCGCCGAAAGATC<br>GGCCCGCGTCTGATTAGCTAGTTGGTGAGGTAATGGCTCACCAAGGCGACGATCAGTA<br>GCTGGTCTGAGAGGATGATCAGCCACATTGGGACTGAGACACGGCCCAAACTCCTACG<br>GGAGGCAGCAGTGGGGAATATTGGACAATGGGGGAAACCCTGATCCAGCCATGCCGC<br>GTGAGTGATGAAGGCCCTAGGGTTGTAAAGCTCTTTTGTGCGGGAAGATAATGACGGT<br>ACCGCAAGAATAAGCCCCGGCTAACTTCGTGCCAGCAGCCGCGGTAATACGAAGGGGG<br>CTAGCGTTGCTCGGAATCACTGGGCGTAAAGGGTGCGTAGGCGGGTTTCTAAGTCAGA<br>GGTGAAAGCCTGGAGCTCAACTCCAGAACTGCCTTTGATACTGGAAGTCTTGAGTATG<br>GCAGAGGTGAGTGGAACTGCGAGTGTAGAGGTGAAATTCGTAGATATTCGCAAGAACA<br>CCAGTGGCGAAGGCGGCTCACTGGGCCATTACTGACGCTGAGGCACGAAAGCGTGGGG<br>AGCAAACA
<Complete gene sequence (1,411 bp)><br><br>AGCGAACGCTGGCGGCAGGCTTAACACATGCAAGTCGAACGGGCGTAGCAATACGTCA<br>GTGGCAGACGGGTGAGTAACGCGTGGGAACGTACCTTTTGGTTCGGAACAACACAGGG<br>AAACTTGTGCTAATACCGGATAAGCCCTTACGGGGAAAGATTTATCGCCGAAAGATCG<br>GCCCGCGTCTGATTAGCTAGTTGGTGAGGTAATGGCTCACCAAGGCGACGATCAGTAG<br>CTGGTCTGAGAGGATGATCAGCCACATTGGGACTGAGACACGGCCCAAACTCCTACGG<br>GAGGCAGCAGTGGGGAATATTGGACAATGGGGGAAACCCTGATCCAGCCATGCCGCGT<br>GAGTGATGAAGGCCCTAGGGTTGTAAAGCTCTTTTGTGCGGGAAGATAATGACGGTAC<br>CGCAAGAATAAGCCCCGGCTAACTTCGTGCCAGCAGCCGCGGTAATACGAAGGGGGCT<br>AGCGTTGCTCGGAATCACTGGGCGTAAAGGGTGCGTAGGCGGGTTTCTAAGTCAGAGG<br>TGAAAGCCTGGAGCTCAACTCCAGAACTGCCTTTGATACTGGAAGTCTTGAGTATGGC<br>AGAGGTGAGTGGAACTGCGAGTGTAGAGGTGAAATTCGTAGATATTCGCAAGAACACC<br>AGTGGCGAAGGCGGCTCACTGGGCCATTACTGACGCTGAGGCACGAAAGCGTGGGGA<br>GCAAACAGGATTAGATACCCTGGTAGTCCACGCCGTAAACGATGAATGCCAGCCGTTA<br>GTGGGTTTACTCACTAGTGGCGCAGCTAACGCTTTAAGCATTCCGCCTGGGGAGTACGG<br>TCGCAAGATTAAAACTCAAAGGAATTGACGGGGGCCCGCACAAGCGGTGGAGCATGT<br>GGTTTAATTCGACGCAACGCGCAGAACCTTACCAGCCCTTGACATGTCCAGGACCGGT<br>CGCAGAGACGCGACCTTCTCTTCGGAGCCTGGAGCACAGGTGCTGCATGGCTGTCGTC<br>AGCTCGTGTCGTGAGATGTTGGGTTAAGTCCCGCAACGAGCGCAACCCCCGTCCTTAGT<br>TGCTACCATTTAGTTGAGCACTCTAAGGAGACTGCCGGTGATAAGCCGCGAGGAAGGT<br>GGGGATGACGTCAAGTCCTCATGGCCCTTACGGGCTGGGCTACACACGTGCTACAATG<br>GCGGTGACAATGGGAAGCTAAGGGGCGACCCTTCGCAAATCTCAAAAAGCCGTCTCAG<br>TTCGGATTGGGCTCTGCAACTCGAGCCCATGAAGTTGGAATCGCTAGTAATCGTGGATC<br>AGCATGCCACGGTGAATACGTTCCCGGGCCTTGTACACACCGCCCGTCACACCATGGG<br>AGTTGGCTTTACCTGAAGACGGTGCGCTAACCAGCAATGGGGGCAGCCGGCCACGGTA<br>GGGTCAGCGACTGGGGTG

Table S3. CBCs and biochemical parameters of the mice injected with PBS or AUN dispersion after 30 days.

Measured value	Entry	Unit	PBS (n = 5)	AUN (n = 5)	P value
CBC	WBC	$\times 10^2$ μL^{-1}	56.00 ± 7.07	56.33 ± 7.57	> 0.05
	RBC	$\times 10^4$ μL^{-1}	865.80 ± 19.25	876.20 ± 50.79	> 0.05
	HGB	g dL^{-1}	12.58 ± 0.37	12.66 ± 0.81	> 0.05
	HCT	%	40.74 ± 0.79	40.82 ± 2.40	> 0.05
	MCV	fL	47.08 ± 0.71	46.58 ± 0.53	> 0.05
	MCH	pg	14.52 ± 0.26	14.44 ± 0.15	> 0.05
	MCHC	g dL^{-1}	30.09 ± 0.49	31.00 ± 0.33	> 0.05
	PLT	$\times 10^4$ μL^{-1}	67.46 ± 4.97	68.10 ± 5.05	> 0.05
Biochemical parameters	TP	g dL^{-1}	4.10 ± 0.13	4.00 ± 0.18	> 0.05
	ALB	g dL^{-1}	2.90 ± 0.09	2.70 ± 0.13	> 0.05
	BUN	mg dL^{-1}	21.02 ± 3.61	21.94 ± 2.77	> 0.05
	CRE	mg dL^{-1}	0.10 ± 0.01	0.10 ± 0.01	> 0.05
	Na	mEq L^{-1}	151.40 ± 1.34	152.40 ± 1.52	> 0.05
	K	mEq L^{-1}	3.38 ± 0.28	3.14 ± 0.36	> 0.05
	Cl	mEq L^{-1}	118.20 ± 1.10	118.80 ± 0.84	> 0.05
	AST	IU L^{-1}	64.00 ± 8.49	67.60 ± 11.37	> 0.05
	ALT	IU L^{-1}	34.50 ± 4.95	35.40 ± 3.98	> 0.05
	LDH	IU L^{-1}	284.25 ± 60.32	264.80 ± 56.14	> 0.05
	AMY	IU L^{-1}	1806.00 ± 157.14	1853.20 ± 193.67	> 0.05
	CK	IU L^{-1}	211.50 ± 50.35	205.67 ± 71.46	> 0.05

Data are represented as the mean ± SEM; n = 5 biologically independent mice. Statistical analyses were performed using the Student's two-sided t-test.
Abbreviations: ALB, albumin; ALT, alanine transaminase; AMY, amylase; AST, aspartate aminotransferase; BUN, blood urea nitrogen; Cl, chlorine; CK, creatine kinase; CRE, creatinine; CRP, C-reactive protein; HCT, hematocrit; HGB, hemoglobin; K, potassium; LDH, lactate dehydrogenase; MCH, mean corpuscular hemoglobin; MCHC, mean corpuscular hemoglobin concentration; MCV, mean corpuscular volume; Na, sodium; PLT, platelet; RBC, red blood cell; TP, total protein; WBC, white blood cell.

Table S4. CBCs and biochemical parameters of the mice injected with PBS or A-gyo dispersion after 30 days.

Measured value	Entry	Unit	PBS (n = 5)	A-gyo (n = 5)	P value
CBC	WBC	$\times 10^2\ \mu L^{-1}$	66.2 ± 12.70	66.8 ± 12.13	> 0.05
	RBC	$\times 10^4\ \mu L^{-1}$	948.8 ± 8.70	883.8 ± 31.58	> 0.05
	HGB	$g\ dL^{-1}$	14.1 ± 0.31	13.88 ± 0.46	> 0.05
	HCT	%	44.7 ± 0.91	40.98 ± 1.67	> 0.05
	MCV	fL	47.1 ± 0.81	46.38 ± 0.27	> 0.05
	MCH	pg	14.8 ± 0.19	15.7 ± 0.12	> 0.05
	MCHC	$g\ dL^{-1}$	31.5 ± 0.40	33.84 ± 0.24	> 0.05
	PLT	$\times 10^4\ \mu L^{-1}$	72.6 ± 4.67	70.76 ± 6.38	> 0.05
Biochemical parameters	TP	$g\ dL^{-1}$	4.1 ± 0.15	3.9 ± 0.16	> 0.05
	ALB	$g\ dL^{-1}$	2.7 ± 0.15	2.6 ± 0.06	> 0.05
	BUN	$mg\ dL^{-1}$	21.5 ± 2.04	22.0 ± 1.85	> 0.05
	CRE	$mg\ dL^{-1}$	0.13 ± 0.01	0.12 ± 0.03	> 0.05
	Na	$mEq\ L^{-1}$	153.0 ± 1.14	151.0 ± 0.55	> 0.05
	K	$mEq\ L^{-1}$	3.6 ± 0.42	3.6 ± 0.14	> 0.05
	Cl	$mEq\ L^{-1}$	117.2 ± 0.45	117.8 ± 1.30	> 0.05
	AST	$IU\ L^{-1}$	51.4 ± 6.43	58.8 ± 10.43	> 0.05
	ALT	$IU\ L^{-1}$	33.2 ± 7.36	34.6 ± 6.58	> 0.05
	LDH	$IU\ L^{-1}$	244.2 ± 75.88	254.6 ± 68.68	> 0.05
	AMY	$IU\ L^{-1}$	1671.4 ± 97.66	1682.8 ± 131.58	> 0.05
	CK	$IU\ L^{-1}$	181.6 ± 113.95	182.6 ± 128.41	> 0.05

Data are represented as the mean ± SEM; n = 5 biologically independent mice. Statistical analyses were performed using the Student's two-sided t-test.
Abbreviations: ALB, albumin; ALT, alanine transaminase; AMY, amylase; AST, aspartate aminotransferase; BUN, blood urea nitrogen; Cl, chlorine; CK, creatine kinase; CRE, creatinine; CRP, C-reactive protein; HCT, hematocrit; HGB, hemoglobin; K, potassium; LDH, lactate dehydrogenase; MCH, mean corpuscular hemoglobin; MCHC, mean corpuscular hemoglobin concentration; MCV, mean corpuscular volume; Na, sodium; PLT, platelet; RBC, red blood cell; TP, total protein; WBC, white blood cell.

Table S5. Obtained 16S rRNA gene sequences of i-LS.

<Partial gene sequence (764 bp)>

GGACGAACGCTGGCGGCGTGCCTAATACATGCAAGTCGAGCGACGAATCGAGGTACTT
GTACCAAGACGAAGAGCGGCGAACGGGTGAGTAACGCGTGGGAAATCTGCCGAGTAG
CGGGGGACAACGTTTGGAAACGAACGCTAATACCGCATAACAATTGGAATCGCATGAT
TCTTATTTAAAAGAAGCAAAAGCTTCACTACTTGATGATCCCGCGTTGTATTAGCTAGT
TGGTAGTGTAAAGGACTACCAAGGCGATGATACATAGCCGGCCTGAGAGGGTGAACG
GCCACACTGGGACTGAGACACGGCCCAGACTCCTACGGGAGGCAGCAGTAGGGAATCT
TCGGCAATGGACGAAAGTCTGACCGAGCAACGCCGCGTGAGTGAAGAAGGTTTTCGGA
TCGTAAAACTCTGTTGTTAGAGAAGAACGTTAAGTAGAGTGGAAAGTTACTTAAGTGA
CGGTATCTAACCAGAAAGGGACGGCTAACTACGTGCCAGCAGCCGCGGTAATACGTAG
GTCCCGAGCGTTGTCCGGATTTATTGGGCGTAAAGCGAGCGCAGGTGGTTTCTTAAGTC
TGATGTAAAAGGCAGTGGCTCAACCATTGTGTGCATTGGAAACTGGGGAACTTGAGTG
CAGGAGAGGGGAGTGGAATTCCATGTGTAGCGGTGAAATGCGTAGATATATGGAGGA
ACACCGGAGGCGAAAGCGGCTCTCTGGCCTGTAACTGACACTGAGGCTCGAAAGCGTG
GGGAGCAAACA

<Complete gene sequence (1,469 bp)>

GACGAACGCTGGCGGCGTGCCTAATACATGCAAGTCGAGCGACGAATCGAGGTACTTG
TACCAAGACGAAGAGCGGCGAACGGGTGAGTAACGCGTGGGAAATCTGCCGAGTAGC
GGGGGACAACGTTTGGAAACGAACGCTAATACCGCATAACAATTGGAATCGCATGATT
CTTATTTAAAAGAAGCAAAAGCTTCACTACTTGATGATCCCGCGTTGTATTAGCTAGTT
GGTAGTGTAAAGGACTACCAAGGCGATGATACATAGCCGGCCTGAGAGGGTGAACGG
CCACACTGGGACTGAGACACGGCCCAGACTCCTACGGGAGGCAGCAGTAGGGAATCTT
CGGCAATGGACGAAAGTCTGACCGAGCAACGCCGCGTGAGTGAAGAAGGTTTTCGGAT
CGTAAAACTCTGTTGTTAGAGAAGAACGTTAAGTAGAGTGGAAAGTTACTTAAGTGAC
GGTATCTAACCAGAAAGGGACGGCTAACTACGTGCCAGCAGCCGCGGTAATACGTAGG
TCCCGAGCGTTGTCCGGATTTATTGGGCGTAAAGCGAGCGCAGGTGGTTTCTTAAGTCT
GATGTAAAAGGCAGTGGCTCAACCATTGTGTGCATTGGAAACTGGGGAACTTGAGTGC
AGGAGAGGGGAGTGGAATTCCATGTGTAGCGGTGAAATGCGTAGATATATGGAGGAA
CACCGGAGGCGAAAGCGGCTCTCTGGCCTGTAACTGACACTGAGGCTCGAAAGCGTGG
GGAGCAAACAGGATTAGATACCCTGGTAGTCCACGCCGTAAACGATGAGTGCTAGCTG
TAGGGAGCTATAAGTTCTCTGTAGCGCAGCTAACGCATTAAGCACTCCGCCTGGGGAG
TACGACCGCAAGGTTGAAACTCAAAGGAATTGACGGGGGCCCGCACAAGCGGTGGAG
CATGTGGTTTAATTCGAAGCAACGCGAAGAACCTTACCAGGTCTTGACATCCCGATGC
AATCCTTAGAGATAAGGAGTTACTTCGGTACATCGGTGACAGGTGGTGCATGGTTGTC
GTCAGCTCGTGTCGTGAGATGTTGGGTTAAGTCCCGCAACGAGCGCAACCCTTATTACT
AGTTGCCATCATTAAGTTGGGCACTCTAGTGAGACTGCCGGTGATAAACCGGAGGAAG
GTGGGGATGACGTCAAATCATCATGCCCCTTATGACCTGGGCTACACACGTGCTACAAT
GGGTGGTACAACGAGTCGCCAACCCGCGAGGGTGCGCTAATCTCTTAAAACCATTCTC
AGTTCGGATTGCAGGCTGCAACTCGCCTGCATGAAGTCGGAATCGCTAGTAATCGCGG
ATCAGCACGCCGCGGTGAATACGTTCCCGGGCCTTGTACACACCGCCCGTCACACCAC
GGAAGTTGGGAGTACCCAAAGTAGGTTGCCTAACCGCAAGGAGGGCGCTTCCTAAGGT
AAGACCGATGACTGGGGTG

Table S6. Obtained 16S rRNA gene sequences of i-EF

<Partial gene sequence (776 bp)><br><br>GGACGAACGCTGGCGGCGTGCCTAATACATGCAAGTCGAACGCTTCTTTCCTCCCGAGT<br>GCTTGCACTCATTTGGAAAGAGGAGTGGCGGACGGGTGAGTAACACGTGGGTAACCTA<br>CCCATCAGAGGGGGATAACACTTGGAAACAGGTGCTAATACCGCATAACAGTTTATGC<br>CGCATGGCATAAGAGTGAAAGGCGCTTTCGGGTGTCACTGATGGATGGACCCGCGGTG<br>CATTAGCTAGTTGGTGAGGTAACGGCTCACCAAGGCCACGATGCATAGCCGACCTGAG<br>AGGGTGATCGGCCACACTGGGACTGAGACACGGCCCAGACTCCTACGGGAGGCAGCA<br>GTAGGGAATCTTCGGCAATGGACGAAAGTCTGACCGAGCAACGCCGCGTGAGTGAAG<br>AAGGTTTTCGGATCGTAAAACTCTGTTGTTAGAGAAGAACAAGGACGTTAGTAACTGA<br>ACGTCCCCTGACGGTATCTAACCAGAAAGCCACGGCTAACTACGTGCCAGCAGCCGCG<br>GTAATACGTAGGTGGCAAGCGTTGTCCGGATTTATTGGGCGTAAAGCGAGCGCAGGCG<br>GTTTCTTAAGTCTGATGTGAAAGCCCCCGGCTCAACCGGGGAGGGTCATTGGAAACTG<br>GGAGACTTGAGTGCAGAAGAGGAGAGTGGAATTCCATGTGTAGCGGTGAAATGCGTA<br>GATATATGGAGGAACACCAGTGGCGAAGGCGGCTCTCTGGTCTGTAACTGACGCTGAG<br>GCTCGAAAGCGTGGGGAGCAAACA
<Complete gene sequence (1,483 bp)><br><br>GACGAACGCTGGCGGCGTGCCTAATACATGCAAGTCGAACGCTTCTTTCCTCCCGAGTG<br>CTTGCACTCATTTGGAAAGAGGAGTGGCGGACGGGTGAGTAACACGTGGGTAACCTAC<br>CCATCAGAGGGGGATAACACTTGGAAACAGGTGCTAATACCGCATAACAGTTTATGCC<br>GCATGGCATAAGAGTGAAAGGCGCTTTCGGGTGTCACTGATGGATGGACCCGCGGTGC<br>ATTAGCTAGTTGGTGAGGTAACGGCTCACCAAGGCCACGATGCATAGCCGACCTGAGA<br>GGGTGATCGGCCACACTGGGACTGAGACACGGCCCAGACTCCTACGGGAGGCAGCAGT<br>AGGGAATCTTCGGCAATGGACGAAAGTCTGACCGAGCAACGCCGCGTGAGTGAAGAA<br>GGTTTTCGGATCGTAAAACTCTGTTGTTAGAGAAGAACAAGGACGTTAGTAACTGAAC<br>GTCCCCTGACGGTATCTAACCAGAAAGCCACGGCTAACTACGTGCCAGCAGCCGCGGT<br>AATACGTAGGTGGCAAGCGTTGTCCGGATTTATTGGGCGTAAAGCGAGCGCAGGCGGT<br>TTCTTAAGTCTGATGTGAAAGCCCCCGGCTCAACCGGGGAGGGTCATTGGAAACTGGG<br>AGACTTGAGTGCAGAAGAGGAGAGTGGAATTCCATGTGTAGCGGTGAAATGCGTAGAT<br>ATATGGAGGAACACCAGTGGCGAAGGCGGCTCTCTGGTCTGTAACTGACGCTGAGGCT<br>CGAAAGCGTGGGGAGCAAACAGGATTAGATACCCTGGTAGTCCACGCCGTAAACGATG<br>AGTGCTAAGTGTTGGAGGGTTTCCGCCCTTCAGTGCTGCAGCAAACGCATTAAGCACTC<br>CGCCTGGGGAGTACGACCGCAAGGTTGAAACTCAAAGGAATTGACGGGGGCCCGCAC<br>AAGCGGTGGAGCATGTGGTTTAATTCGAAGCAACGCGAAGAACCTTACCAGGTCTTGA<br>CATCCTTTGACCACTCTAGAGATAGAGCTTTCCCTTCGGGGACAAAGTGACAGGTGGTG<br>CATGGTTGTCGTCAGCTCGTGTCGTGAGATGTTGGGTTAAGTCCCGCAACGAGCGCAAC<br>CCTTATTGTTAGTTGCCATCATTTAGTTGGGCACTCTAGCGAGACTGCCGGTGACAAAC<br>CGGAGGAAGGTGGGGATGACGTCAAATCATCATGCCCCTTATGACCTGGGCTACACAC<br>GTGCTACAATGGGAAGTACAACGAGTCGCTAGACCGCGAGGTCATGCAAATCTCTTAA<br>AGCTTCTCTCAGTTCGGATTGCAGGCTGCAACTCGCCTGCATGAAGCCGGAATCGCTAG<br>TAATCGCGGATCAGCACGCCGCGGTGAATACGTTCCCGGGCCTTGTACACACCGCCCG<br>TCACACCACGAGAGTTTGTAACACCCGAAGTCGGTGAGGTAACCTTTTTGGAGCCAGC<br>CGCCTAAGGTGGGATAGATGATTGGGGTG

Table S7. Primers used for amplification and sequencing of the 16S rRNA gene of isolated bacteria.

Object	Primer	Sequence
Primers for analyzing partial gene sequences	10F<br>800R	5'-GTTTGATCCTGGCTCA-3'<br>5'-TACCAGGGTATCTAATCC-3'
Primers for analyzing complete gene sequences	27F<br>1492R	5'-AGAGTTTGATCCTGGCTCAG-3'<br>5'-GGCTACCTTGTTACGACTT-3'

Table S8. Antibodies used in this study.

**Antibody**	**Type**	**Source**	**Catalog No.**	**Application**
CD4	Rabbit Monoclonal	Cell Signaling Technology	25229	IHC (1:100)
CD8	Rabbit Monoclonal	Cell Signaling Technology	98941	IHC (1:200)
F4/80	Mouse Monoclonal	BMA Biomedicals	T-2028	IHC (1:50)
CD3	Rabbit Monoclonal	Abcam	ab16669	IHC (1:100)
CD19	Rabbit Polyclonal	Bioss	bs-0079R	IHC (1:100)
CXCR4	Goat Polyclonal	Abcam	ab1670	IHC (1:100)
NKp46	Rabbit Polyclonal	Affinity Biosciences	DF7599	IHC (1:100)
Caspase-3	Rabbit Polyclonal	Cell Signaling Technology	9661S	IHC (1:100)
TNF-α	Rabbit Polyclonal	Abcam	ab6671	IHC (1:100)
Anti-digoxigenin-peroxidase	Sheep Polyclonal	Merck Millipore	S7100	Tunel
FITC–CD4	Rat Monoclonal	BioLegend	100406	Flow cytometry
FITC–CD8a	Rat Monoclonal	BioLegend	100706	Flow cytometry
FITC–CD45RO	Mouse Monoclonal	BioLegend	304242	Flow cytometry
FITC–F4/80	Rat Monoclonal	BioLegend	123108	Flow cytometry
FITC–CD3	Rat Monoclonal	BioLegend	100204	Flow cytometry
FITC–CD19	Rat Monoclonal	BioLegend	152404	Flow cytometry
Alexa Fluor 488–CD184 (CXCR4)	Rat Monoclonal	Thermo Fisher Scientific	53-9991-80	Flow cytometry
FITC–NK-1.1	Mouse Monochlonal	BioLegend	108706	Flow cytometry

Table S9. Probes used in microbial FISH analysis.

Probe name	Accession no.	Specificity	Source	Target rRNA	Sequence
Rhodopseud	pB-1634	*Rhodopseudomonas*	Chromosome Science Labo	16S rRNA	5'-GACTTAGAAACCCGCCTACG-3'
EUB338	-	*Proteus mirabilis*	Chromosome Science Labo	16S rRNA	5'-GCCCCTGCTTTGGTC-3'
